# LC-ESI-QTOF-MS/MS Characterization of Seaweed Phenolics and Their Antioxidant Potential

**DOI:** 10.3390/md18060331

**Published:** 2020-06-24

**Authors:** Biming Zhong, Nicholas A. Robinson, Robyn D. Warner, Colin J. Barrow, Frank R. Dunshea, Hafiz A.R. Suleria

**Affiliations:** 1 School of Agriculture and Food, Faculty of Veterinary and Agricultural Sciences, The University of Melbourne, Parkville, VIC 3010, Australia; bimingz@student.unimelb.edu.au (B.Z.); robyn.warner@unimelb.edu.au (R.D.W.); fdunshea@unimelb.edu.au (F.R.D.); 2 Sustainable Aquaculture Laboratory-Temperate and Tropical (SALTT), School of BioSciences, The University of Melbourne, Parkville, VIC 3010, Australia; nicholas.robinson@nofima.no; 3 Norwegian Institute of Food, Fisheries and Aquaculture Research (Nofima), NO-1431 Ås, Norway; 4 Centre for Chemistry and Biotechnology, School of Life and Environmental Sciences, Deakin University, Waurn Ponds, VIC 3217, Australia; colin.barrow@deakin.edu.au

**Keywords:** seaweeds, polyphenols, antioxidant potential, LC-ESI-QTOF-MS/MS, HPLC-PDA

## Abstract

Seaweed is an important food widely consumed in Asian countries. Seaweed has a diverse array of bioactive compounds, including dietary fiber, carbohydrate, protein, fatty acid, minerals and polyphenols, which contribute to the health benefits and commercial value of seaweed. Nevertheless, detailed information on polyphenol content in seaweeds is still limited. Therefore, the present work aimed to investigate the phenolic compounds present in eight seaweeds [Chlorophyta (green), *Ulva* sp., *Caulerpa* sp. and *Codium* sp.; Rhodophyta (red), *Dasya* sp., *Grateloupia* sp. and *Centroceras* sp.; Ochrophyta (brown), *Ecklonia* sp., *Sargassum* sp.], using liquid chromatography electrospray ionization quadrupole time-of-flight mass spectrometry (LC-ESI-QTOF-MS/MS). The total phenolic content (TPC), total flavonoid content (TFC) and total tannin content (TTC) were determined. The antioxidant potential of seaweed was assessed using a 2,2-diphenyl-1-picrylhydrazyl (DPPH) free radical scavenging assay, a 2,2′-azino-bis-3-ethylbenzothiazoline-6-sulfonic acid (ABTS) free radical scavenging assay and a ferric reducing antioxidant power (FRAP) assay. Brown seaweed species showed the highest total polyphenol content, which correlated with the highest antioxidant potential. The LC-ESI-QTOF-MS/MS tentatively identified a total of 54 phenolic compounds present in the eight seaweeds. The largest number of phenolic compounds were present in *Centroceras* sp. followed by *Ecklonia* sp. and *Caulerpa* sp. Using high-performance liquid chromatography-photodiode array (HPLC-PDA) quantification, the most abundant phenolic compound was *p*-hydroxybenzoic acid, present in *Ulva* sp. at 846.083 ± 0.02 μg/g fresh weight. The results obtained indicate the importance of seaweed as a promising source of polyphenols with antioxidant properties, consistent with the health potential of seaweed in food, pharmaceutical and nutraceutical applications.

## 1. Introduction

Seaweed has been utilized as a food for humans for centuries, and the current global market is valued at more than USD 6 billion per annum with an annual volume of approximately 12 million tonnes in 2018 [1,2]. Seaweeds (macroalgae) are classified into three major groups including Chlorophyta (green algae), Rhodophyta (red algae) and Ochrophyta (brown algae) based on their color. It is estimated that 1800 different green macroalgae, 6200 red macroalgae, and 1800 brown macroalgae are found in the marine environment [3]. Like plants, they have chlorophyll for photosynthesis but also contain other pigments which may be colored red, blue, brown or gold. Seaweeds are used in many countries as a source of food especially in East Asia, seaweeds are associated with different Japanese, Koreans and Chinese cuisines [4]. Seaweed is considered an excellent source of bioactive compounds with positive health effects, including carotenoids, phenolics, chitosan, gelatin, polyunsaturated fatty acids, various vitamins and minerals [5]. Recent interest in seaweed has focused on seaweed natural bioactive compounds in the functional food, pharmaceutical and cosmeceutical industries [6]. Among these bioactives, polyphenols, which are defined as the compounds containing one or more aromatic rings bearing hydroxyl groups, have attracted considerable attention [7]. Polyphenols have been shown to exhibit antioxidant, antimicrobial, antidiabetic, anti-inflammatory and anticancer properties in in vitro and in vivo studies [8], and are categorized into subclasses of phenolic acids, flavonoids, stilbenes, and lignans, depending on the chemical structure [9]. 

A promising bioactive property of polyphenols relates to their antioxidant activity and redox potential, allowing them to reduce the reactive oxygen species (ROS) that are involved in a range of human disorders [10]. Strong antioxidant properties of various edible seaweeds have been reported, particularly with seaweeds with high polyphenol content, which can be as high as 20–30% of the dry weight of some brown seaweeds [11,12]. Several phenolic compounds are abundant in a range of species of seaweed, including gallic acid, protocatechuic acid, caffeic acid and epicatechin, with these species showing potential as functional foods [13]. Antioxidants in food can exhibit their activity by donating hydrogen atoms, providing electrons and chelating free metals [14]. Antioxidant compounds have been successfully extracted from seaweeds and commercialized for their health benefits or for their ability to prolong the shelf-life of food through their antioxidant potential [15,16]. 

Total phenolic, flavonoid and tannin contents in seaweed can be indirectly measured using assays for total phenolic content (TPC), total flavonoid content (TFC) and total tannin (TTC), respectively. The antioxidant activities of seaweed can be quantified using various assays based on different mechanisms, including 2,2-diphenyl-1-picrylhydrazyl (DPPH) and 2,2′-azino-bis-3-ethylbenzothiazoline-6-sulfonic acid (ABTS) assays based on free-radical scavenged by antioxidant compounds, and ferric reducing of antioxidant power (FRAP) assay based on the reducing capacity of antioxidants [17]. However, TPC and other colorimetric methods neither separate, nor quantify, individual compounds. High-performance liquid chromatography coupled with electrospray ionization-quadrupole-time of flight-mass spectrometry (LC-ESI-QTOF-MS/MS) has been a standard method to isolate and characterize phenolic compounds based on their molecular weight [18]. High-performance liquid chromatography photodiode array (HPLC-PDA) has been used to quantify various bioactive compounds in seaweed extracts [19].

The objectives of the current study were: (1) to extract phenolic compounds from a range of seaweeds; (2) quantify the total phenolic and antioxidant capacities of seaweed extracts using different assays and (3) apply LC-ESI-QTOF-MS/MS and HPLC-PDA to characterize and quantify individual phenolic compounds.

## 2. Results and Discussion

### 2.1. Polyphenol Estimation (TPC, TFC and TTC)

The polyphenol content was measured as TPC, TFC and TTC (Table 1). Brown seaweed *Ecklonia* sp. showed significantly higher TPC (1044 ± 2.5 μg GAE/g_f.w._) and TTC (167 ± 23.2 μg CE/g_f.w._) contents than other seaweed (*p* < 0.05). The presence of higher total phenolics in brown seaweed *Ecklonia* sp. compared to green seaweed *Ulva* sp. and red seaweed *Porphyra* sp. was previously observed by García-Casal, et al. [20]. The significant higher total phenolic and tannin content in brown seaweed *Ecklonia* sp. is proposed to be related to the presence of phlorotannins, which are restricted to brown algae, in special vesicles (physodes) within the cells [21]. Phlorotannins are highly complex compounds formed by the polymerization of phloroglucinol, which has already been characterized by LC-MS in previous studies [22,23] and supported by our current study. The highest total flavonoid content was found in red seaweed *Grateloupia* sp. (54.4 ± 0.74 μg QE/g_f.w._) (*p* < 0.05) as compared to brown and green seaweeds. However, compared to previous studies [24], the total flavonoid content of red seaweed we found was relatively low compared with that of brown and green seaweed. The inconsistency might be explained by Chan, et al. [25], who reported that the total flavonoid content of seaweeds is impacted by sunlight, climate, region and extraction solvent.

Regarding seaweed groups, brown seaweeds presented statistically higher TPC and TTC values than green and red seaweeds (*p* < 0.05). This is in agreement with previous research which reported that brown seaweed had a higher total phenolic content than red and green seaweeds [26]. In addition, a study conducted by Cox, Abu-Ghannam and Gupta [24] also indicated that the total tannin content of brown seaweeds was significantly higher than that of green and red seaweed, which is explained by the presence of the unique polyphenolic components of phlorotannin in brown seaweed [27].

### 2.2. Antioxidant Activities (ABTS, DPPH and FRAP)

The antioxidant activities were determined using ABTS, DPPH and FRAP assays (Table 2.). The brown seaweed *Ecklonia* sp. had a significantly higher level of antioxidant potential than other seaweeds (958 ± 0.4 μg AAE/g_f.w._ for ABTS, 510 ± 3.4 μg AAE/g_f.w._ for DPPH and 170 ± 2.0 μg AAE/g_f.w._ for FRAP, *p* < 0.05). The result was consistent with a previous study where phlorotannins were successfully isolated from *Ecklonia* sp. and exhibited strong DPPH radical scavenging activity [28]. In the present work, although *Ulva* sp., *Caulerpa* sp. and *Codium* sp. exhibited ABTS radical scavenging activities, no DPPH radical scavenging activities were detected. This might be due to limitations of the DPPH assay [29]. Firstly, unlike water-soluble ABTS^+^, hydrophobic DPPH must be performed in organic solvent, which interferes with the hydrogen atom transfer reaction by disturbing the release of hydrogen atoms. Secondly, DPPH reacts rapidly, mainly through single electron transfer, with ascorbic acid and simple phenols with no ring adducts, but slowly with complex phenolic compounds with side chains and ring adducts. Therefore, the application of organic solvent and the complex structure of phenolic compounds in seaweed might lead to underestimation of DPPH scavenging activities. 

Within the seaweed groups, brown seaweed species presented significantly higher antioxidant properties for all assays than green and red seaweed species (*p* < 0.05). This result was in accordance with a previous study, which also found brown seaweed had higher ABTS radical scavenging activity than red or green seaweeds [30].

The relationship between TPC and antioxidant potential of all three type of (green, red and brown) seaweeds was confirmed by performing a regression model between the values of TPC and each antioxidant assay. Results showed a significant positive correlation between TPC and antioxidant activity (r^2^ = 0.926 for ABTS, r^2^ = 0.714 for DPPH and r^2^ = 0.899 for FRAP, *p* < 0.05). A positive correlation between total phenolic content and antioxidant assay results was also supported by previous studies, suggesting that phenolics are the major contributor to the excellent antioxidant properties of seaweeds [21,30].

### 2.3. LC-ESI-QTOF-MS/MS Characterization of The Phenolic Compounds

LC-MS has been widely used for the characterization of the phenolic profiles of different plant and marine samples [31]. A qualitative analysis of the phenolic compounds from different seaweed extracts were achieved by LC-ESI-QTOF-MS/MS analysis in negative and positive ionization modes (Table S1, Figures S1 and S2-Supplementary Materials). Phenolic compounds present in eight different seaweeds were tentatively identified from their *m/z* value and MS spectra in both negative and positive ionization modes ([M − H]^−^/[M + H]^+^) using Agilent LC-MS Qualitative Software and Personal Compound Database and Library (PCDL). Compounds with mass error < ± 5 ppm and PCDL library score more than 80 were selected for further MS/MS identification and *m/z* characterization purposes. 

In the present work, LC-MS/MS enabled the tentative identification of 54 phenolic compounds, including 22 phenolic acids, 17 flavonoids, 11 other polyphenols and 4 lignans (Table 3).

#### 2.3.1. Phenolic Acids

Phenolic acids have been reported as the most abundant phenolic compounds in red, green and brown algae [21]. In the present work, four sub-classes of phenolic acid were detected, including hydroxybenzoic acids, hydroxycinnamic acids, hydroxyphenylpentanoic acids and hydroxyphenylacetic acids.

##### Hydroxybenzoic Acids Derivatives

**Figure 1:**
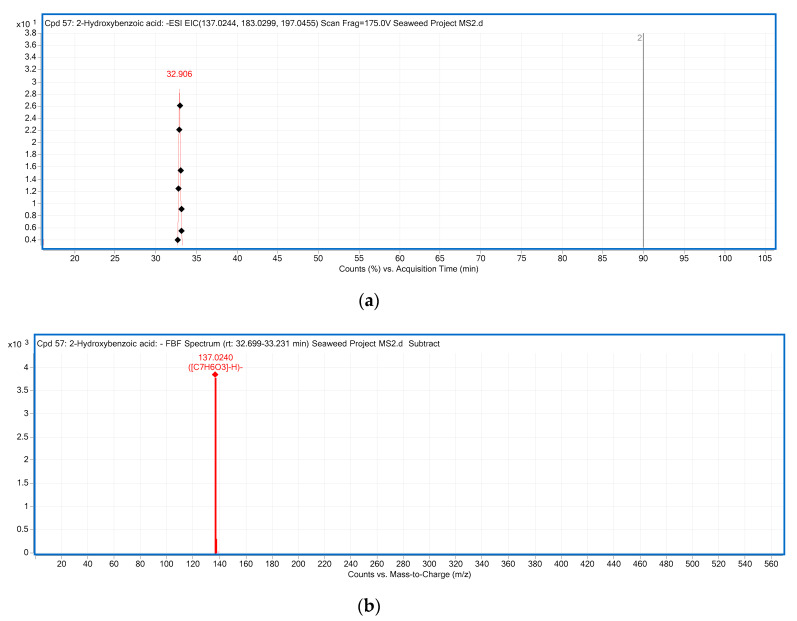

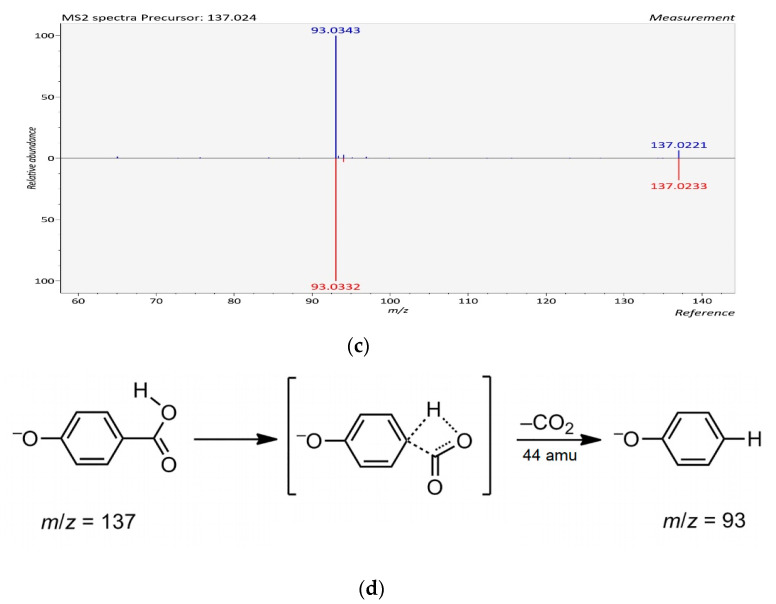
The LC-ESI-QTOF-MS/MS characterization of *p*-hydroxybenzoic acid; (**a**) A chromatograph of *p*-hydroxybenzoic acid (Compound 5, Table 3), Retention time (RT = 32.906 min) in the negative mode of ionization [M − H]^−^ tentatively identified in *Ulva* sp.; (**b**) Mass spectra of *p*-hydroxybenzoic acid with observed/precursor of *m/z* 137.0240 in *Ulva* sp.; (**c**) MS/MS spectrum of *p*-hydroxybenzoic acid reflecting the product ion of *m/z* 93, confirmation via online LC-MS library and database; (**d**) Fragmentation of *p*-hydroxybenzoic acid in negative mode [M − H]^−^, with observed/precursor of *m/z* 137, showing product ion of *m/z* 93 due to the loss of a CO_2_ (44 Da).

Six hydroxybenzoic acid derivatives were detected in six out of eight seaweeds. The typical neutral losses of CO_2_ (44 Da) and hexosyl moiety (162 Da) were observed in phenolic acids [32]. Compound **2** with [M − H]^−^ *m/z* at 169.0138 was only detected from red seaweed *Centroceras* sp., and characterized as gallic acid based on the product ion at 125 *m/z*, corresponding to the loss of CO_2_ (44 Da) from precursor ion [32]. Gallic acid was also previously reported as abundant in the brown seaweed *Himanthalia elongate* [33]. *p*-Hydroxybenzoic acid (Compound **5** with [M − H]^−^ ion at *m/z* 137.0240) present in *Ulva* sp., *Caulerpa* sp. and *Centroceras* sp. was identified and confirmed by MS^2^ experiments (Figure 1). In the MS^2^ spectrum of *m/z* 137.0240, the product ion at *m/z* 93 was due to the loss of a CO_2_ (44 Da) from the parent ion [32]. This is consistent with *p*-hydroxybenzoic acid also being found in seaweeds from the Danish coastal area [34].

4-Hydroxybenzoic acid 4-*O*-glucoside (Compound **3**, *m/z* 299.0778), protocatechuic acid 4-*O*-glucoside (Compound **4**, *m/z* 315.0719) and ellagic acid glucoside (compound **6**, *m/z* 463.0518) were identified in *Sargassum* sp., *Centroceras* sp., *Grateloupia* sp. and *Ecklonia* sp. in both modes. The molecular ions of 4-hydroxybenzoic acid 4-*O*-glucoside, protocatechuic acid 4-*O*-glucoside and ellagic acid glucoside produced the product ions at *m/z* 137, 153 and 301, respectively, indicating the loss of hexosyl moiety (162 Da) from precursor ions [32].

##### Hydroxycinnamic Acids and Other Phenolic Acid Derivatives

Thirteen hydroxycinnamic acids derivatives, two hydroxyphenylpentanoic acids and one hydroxyphenylacetic acid were tentatively identified in our study.

Compound (**7**) was identified as 3-sinapoylquinic acid based on the precursor ion [M − H]^−^ at *m/z* 397.1144, with product ions at *m/z* 223 (sinapic acid ion) and *m/z* 179 (sinapic acid − COO) in *Centroceras* sp. and *Ecklonia* sp., which was previously characterized in extracts of arnica flower [35]. Cinnamoyl glucose (Compound **8**) was also found in *Codium* sp. and *Ulva* sp. The presence of cinnamoyl glucose was confirmed by a [M − H]^−^ *m/z* at 309.0992, which yielded product ions at *m/z* 147, *m/z* 131 and *m/z* 103, indicating the expected loss of hexosyl moiety (162 Da), C_6_H_10_O_6_ (178 Da) and C_7_H_10_O_7_ (206 Da), respectively [36]. 

Compound (**9**), having a precursor ion [M − H]^−^ *m/z* at 341.0882, was tentatively characterized as caffeoyl glucose and was present in *Ecklonia* sp. and *Centroceras* sp. The MS^2^ analysis showed the product ions at *m/z* 179 [M − H − 162] and *m/z* 161 [M − H − 180], consistent with losses of hexosyl moiety and further loss of H_2_O [37]. Compound **14** was tentatively characterized as caffeoyl tartaric acid found in *Grateloupia* sp. and *Centroceras* sp. based on [M − H]^−^ *m/z* at 311.0403. The identification was further supported by the MS^2^ spectrum, which exhibited typical product ion at *m/z* 161, formed by the neutral loss of 150 mass units as a result of tartaric acid fission [38]. To the best of our knowledge, caffeoyl tartaric acid and caffeoyl glucose were previously reported primarily in fruit samples such as grape, however, it was the first time that they were reported in seaweeds [39]. For caffeic acid 3-*O*-glucuronide found in *Caulerpa* sp. (Compound **10** with [M − H]^−^ *m/z* of 355.0671), MS/MS fragmentation yielded the predominant ion at *m/z* 179 after the loss of glucuronide moiety (176 Da), indicating the presence of caffeic acid ion [37].

Compound **11** was tentatively characterized as chlorogenic acid, and only found in *Centroceras* sp. and *Caulerpa* sp. based on [M − H]^−^ *m/z* at 353.0862, and identification was further supported by the MS^2^ spectrum. The identity of chlorogenic acid was confirmed by the product ions at *m/z* 253 [M − H − 100], 190 [M − H − 163] and 144 [M − H − 209], corresponding to the loss of three H_2_O and HCOOH; three H_2_O and C_6_H_5_O_2_; H_2_O and C_7_H_11_O_6_, respectively [40]. Chlorogenic acid was also present in the green seaweed *Capsosiphon fulvescens* from Korea, according to previous research [41].

Four hydroxycinnamic acid derivatives (Compound **12**, **13**, **15** and **17**) were detected in *Caulerpa* sp. in both ionization modes, and were tentatively identified as caffeic acid, caffeic acid 4-sulfate, isoferulic acid 3-sulfate and ferulic acid, according to the precursor ions [M − H]^−^ at *m/z* 179.0350, 258.9929, 273.0086 and 193.0513, respectively. The identification of caffeic acid was confirmed by the product ions at *m/z* 151 [M − H − 28], *m/z* 143 [M − H − 36] and *m/z* 133 [M − H − 46], representing the loss of CO, two H_2_O units and HCOOH, respectively, from the precursor ion [40]. In the MS^2^ experiment of caffeic acid 4-sulfate, the spectra displayed the product ions at *m/z* 179, (presence of caffeic acid ion) and at *m/z* 135, corresponding to the loss of SO_3_ (80 Da) and further loss of CO_2_ (44 Da) from the precursor ion [42]. The similar cleavage was observed in the MS^2^ spectra of isoferulic acid 3-sulfate, which displayed the product ions at *m/z* 193 [M − H − SO_3_] and *m/z* 149 [M − H − SO_3_ − CO_2_], consistent with the presence of isoferulic acid ion (193 Da) and further loss of CO_2_ [42], while the product ions at *m/z* 178 (M − H − 15, loss of CH_3_), *m/z* 149 (M − H − 44, loss of CO_2_) and *m/z* 134 (M − H − 59, loss of CH_3_ and H_2_O) identified ferulic acid [43]. According to a previous study, caffeic acid and ferulic acid were also found in some seaweeds [33,34]. 

Sinapic acid (Compound **16**) were detected in both positive (ESI^+^) and negative (ESI^−^) modes in *Ulva* sp. *Caulerpa* sp. and *Grateloupia* sp. with an observed [M − H]^−^ *m/z* at 223.0621. In the MS^2^ spectrum of sinapic acid, the product ions at *m/z* 205, 179 and 163 were due to the loss of H_2_O (18 Da), CO_2_ (44 Da) and two CH_2_O units (60 Da) from the parent ion, respectively, which was comparable with the fragmentation rules of sinapinic acid [42].

Coumaric acid (compound **18** with [M − H]^−^ *m/z* at 163.0406), yielding a main product ion at *m/z* 119, which corresponded to loss of CO_2_ (44 Da), was found in *Caulerpa* sp. [43]. The presence of coumaric acid in marine seaweeds was also previously reported [34].

Three other phenolic acid derivatives were also detected, including two hydroxyphenylpentanoic acid derivatives and one hydroxyphenylacetic acid derivative. To our best knowledge, this is the first time these other phenolic acid derivatives have been reported in seaweeds. Phenolic acids are the predominant polyphenol compounds found in different seaweeds, which were characterized by using LC-MS in previous studies, and displayed remarkable antioxidant potential [44,45].

#### 2.3.2. Flavonoids

Flavonoid is the main class of phenolic compounds responsible for the antioxidant and free radical scavenging properties observed in seaweed [24]. In the present study, a total of 17 flavonoids were tentatively identified, which were further divided into anthocyanins (03), flavanols (03), flavonols (03), flavone (01) and isoflavonoids (07).

##### Anthocyanins, Flavanols and Flavonols Derivatives

Anthocyanins are naturally occurring pigments that belong to the subclass of flavonoids, which were previously reported in brown Irish seaweeds [46]. In our study, three anthocyanin derivatives were detected only in the red seaweeds *Grateloupia* sp. and *Centroceras* sp., in positive ionization mode. This is the first time all of these anthocyanins derivatives have been reported in seaweeds.

Three flavanols (Compound **26**, **27** and **28**) were detected in all seaweeds except *Centroceras* sp. and *Codium* sp. Compound (**26**) showing precursor ion [M − H]^−^ at *m/z* 305.0668 in negative mode, was the most widely distributed flavanol and was identified as gallocatechin presenting in *Caulerpa* sp., *Ulva* sp., *Dasya* sp., *Ecklonia* sp. and *Sargassum* sp. The presence of gallocatechin derivatives in brown seaweed *ascophyllum nodosum* was reported by Agregán, Munekata, Franco, Dominguez, Carballo and Lorenzo [44] based on the production [M − H]^−^ ion at *m/z* 305. In MS/MS experiment, the product ion at 261 [M − H − 44] was due to the loss of CO_2_ and at *m/z* 219 [M − H − 86] was caused by the loss of C_3_O_2_ and H_2_O [43]. 3′-*O*-methylcatechin (Compound **27** with [M − H]^-^ *m/z* of 303.0886) was identified in *Grateloupia* sp. in the present study, with the product ions at *m/z* 271 (M − H − 32, loss of CH_3_OH) and *m/z* 163 (M − H − 140, loss of CH_3_OH and C_6_H_5_O_2_) [47]. Catechin (isomer) was proposed as compound (**28**), from *Caulerpa* sp., with a precursor ion [M − H]^−^ *m/z* of 289.0731. The MS^2^ spectrum showed the product ions at *m/z* 245, *m/z* 205, and *m/z* 179, indicating the loss of CO_2_ (44 Da), flavonid A ring (84 Da) and flavonid B ring (110 Da) from the precursor ion, respectively [32].

Three flavonols were detected in negative mode in *Centroceras* sp., *Caulerpa* sp. and *Ecklonia* sp. 3,7-dimethylquercetin detected in *Centroceras* sp. was assigned for compound (**31**) based on the observed [M − H]^−^ *m/z* of 329.0674. The further identification of 3,7-dimethylquercetin was achieved by comparing the previous study, which characterized the same compound from *Ipomoea batatas* leaves and showed the product ions at *m/z* 314, *m/z* 299 and *m/z* 271, corresponding to the loss of CH_3_ (15 Da), two CH_3_ (30 Da) and two CH_3_ plus CO unit from the precursor ion, respectively [48].

Rhoifolin (Compound **32** with [M − H]^−^ *m/z* at 577.1588) was the only flavone identified in *Centroceras* sp. with the product ions at *m/z* 413 (M − H − 164) and *m/z* 269 (M − H − 308), representing the loss of rhamnose moiety and H_2_O (164 Da) and hexosyl moiety plus rhamnose moiety (308 Da) from the parent ion [49]. This is the first time that all of the flavonols and flavone derivatives identified in the current study have been reported in seaweeds.

##### Isoflavonoids Derivatives

Isoflavonoids derivatives (a total of seven) were the most diverse flavonoids identified in seaweeds. Sativanone (Compound **33**) was only detected in *Ecklonia* sp. in negative mode with [M − H]^−^ *m/z* at 299.0918. The identity was confirmed by comparing the previous study which characterized sativanone in *Dalbergia odorifera* using LC-MS/MS, and the spectrum displayed the product ions at *m/z* 284 (M − H − 15, loss of CH_3_ from B-ring) and at *m/z* 269 (M − H − 30, loss of two CH_3_) and at *m/z* 225 (M − H − 74, loss of two CH_3_ and a CO_2_) [50]. Compound **37** with [M − H]^-^ *m/z* at 267.0666 exhibited characteristic fragment ions at *m/z* 252 [M − H − CH_3_], *m/z* 224 [M − H − CH_3_ − CO] and *m/z* 180 [M − H − CH_3_ – CO − CO_2_] was identified as dalbergin [50]. To the best of our knowledge, this is the first time that isoflavonoids derivatives were identified and characterized in seaweeds. Flavonoids in different seaweeds with high antioxidant potential have already been reported, which are promising as functional food ingredients or dietary supplements for daily intake [51].

#### 2.3.3. Other Polyphenols

Eleven other polyphenols found were classified as hydroxybenzaldehyde (01), hydroxycoumarins (02), phenolic terpenes (03), tyrosol (02) and other polyphenols (03).

##### Hydroxybenzaldehydes, hydroxycoumarins and hydroxyphenylpropenes Derivatives 

*p*-Hydroxybenzaldehyde (Compound **40** with [M − H]^−^ at *m/z* 121.0295, RT = 15.921 min) was the only hydroxybenzaldehyde presenting in *Dasya* sp., *Ecklonia* sp. and *Codium* sp. The MS^2^ spectrum of *p*-hydroxybenzaldehyde displayed the product ions at *m/z* 92 and *m/z* 77, indicating the loss of CHO (29 Da) and CO_2_ (44 Da) [52]. The presence of *p*-hydroxybenzaldehyde in Irish brown seaweed *Himanthalia elongate* was also previously reported by Rajauria, Foley and Abu-Ghannam [9]. Two hydroxycoumarins derivatives (Compound **41** and **42**) were discovered. Urolithin A with [M − H]^−^ *m/z* at 227.0341 was assigned as compound **41,** from *Grateloupia* sp. MS/MS identification by product ions at *m/z* 198 (M − H − 29, loss of CHO) and 182 *m/z* (M − H − 45, loss of COOH) [53]. Scopoletin with [M − H]^−^ *m/z* at 191.0352 was proposed as compound **42** found in *Codium* sp., *Grateloupia* sp. and *Sargassum* sp., and was identified by the neutral loss of CH_3_ (15 Da) and CO_2_ (44 Da), resulting in product ions at *m/z* 176 and *m/z* 147, respectively [54].

##### Phenolic Terpenes Derivatives

Rosmanol (Compound **43**), showing as precursor ion at [M + H]^+^ at *m/z* 347.1843, was detected in *Dasya* sp., *Ulva* sp., *Grateloupia* sp., *Ecklonia* sp. and *Codium* sp. The product ions at *m/z* 301 and *m/z* 231 came from the loss of a unit of H_2_O and CO (46 Da), and cleavage of molecules pentene, water, and carbon monoxide [55]. Carnosic acid (Compound **45**), identified based on [M − H]^−^ *m/z* at 331.1912, was found in *Ecklonia* sp. *Dasya* sp., *Codium* sp. and *Sargassum* sp. The molecular ion of carnosic acid (*m/z* 331.1912) produced the major fragment ion at *m/z* 287 and *m/z* 269, corresponding to the loss of CO_2_ and further loss of H_2_O from the parent ion [56]. Hermund, et al. [57] also confirmed the presence of carnosic and carnosol as synergistic antioxidants with radical scavenging activity in brown seaweed *Fucus vesiculosus*.

##### Tyrosols and Other Polyphenols Derivatives

Compounds (**46**) were present in *Centroceras* sp., *Dasya* sp., *Grateloupia* sp., and Sargassum, and was tentatively identified as hydroxytyrosol 4-*O*-glucoside based on the observed [M − H]^−^ ions at *m/z* 315.1091. In the MS^2^ spectrum of hydroxytyrosol 4-*O*-glucoside, the typical loss of hexosyl moiety (162 Da) was observed from precursor, resulting in product ions at *m/z* 153 [52]. Compound **47** with [M − H]^−^ *m/z* at 319.1200 was only detected from *Caulerpa* sp., and characterized as 3,4-DHPEA-EDA based on the product ions at *m/z* 301, *m/z* 275 and *m/z* 195, corresponding to loss of H_2_O (18 Da), CO_2_ (44 Da) and C_5_H_6_(CHO)_2_ (124 Da) from the precursor ion [58]. This is the first report of the presence of these tyrosol derivatives in seaweed, while 3,4-DHPEA-AC was previously reported by Gomez-Alonso, et al. [59] in Cornicabra olive oil variety.

Three other polyphenols derivatives were detected, including compound (**49**) with [M − H]^−^ at *m/z* 125.0242, which was proposed as phloroglucinol appearing in brown seaweed *Ecklonia* sp. and *Sargassum* sp. The identity was confirmed by the MS^2^ spectrum, which produced a major fragment ion at *m/z* 97, resulting from the loss of CO (28 Da) from the precursor ion [9]. The presence of phloroglucinol in Irish brown seaweed *Himanthalia elongate* was previously reported by Rajauria, Foley and Abu-Ghannam [9] according to the precursor and product ions, and further confirmed by the UV spectrum and retention time using phloroglucinol standard.

#### 2.3.4. Lignans 

Lignans were minor components present in the seaweeds. In the present study, a total of four lignans were shown to be present in seven out of eight seaweeds.

##### Lignans Derivatives

Compounds **52** detected in *Centroceras* sp. and *Sargassum* sp. was tentatively characterized as arctigenin according to the precursor ions at [M − H]^−^ *m/z* 371.1509. Fragmentation of arctigenin yielded product ions at *m/z* 356, *m/z* 312 and *m/z* 295, corresponding to the loss of CH_3_ (15 Da), unit of CH_3_ and CO_2_ (59 Da), and unit of CH_3_, CO_2_ and OH (76 Da), respectively [60]. Compound **54** (deoxyschisandrin) displaying the [M + H]^+^ *m/z* at 417.2286 and was found in *Ecklonia* sp. and confirmed by the characteristic ions at *m/z* 402 [M − H − CH_3_], *m/z* 347 [M − H − C_5_H_10_], *m/z* 316 [M − H − C_5_H_10_ − OCH_3_] and *m/z* 301 [M − H − C_5_H_10_ − OCH_3_ − CH_3_] [61]. Lignans are abundant in seaweeds, however, the lignans in the present study have not previously been reported in seaweeds [62]. Previously, it was reported that lignans are abundant in seaweeds with various health-promoting properties, including antioxidant, anti-inflammatory and antitumor activities [62,63]. In addition, some epidemiological studies have proposed the therapeutic potential of lignans in chronic diseases, such as cardiovascular disease, type 2 diabetes and cancers [64,65].

The screening and characterization of polyphenolic compounds showed that some of the polyphenols presented in these seaweeds have strong antioxidant potential. Hydroxycinnamic acid derivatives, hydroxybenzoic acids and their derivatives, protocatechuic acid, anthocyanins, flavonoids and their derivatives, hydroxybenzaldehydes, hydroxytyrosol, phloroglucinol and quercetin derivatives are regarded as potential compounds showing considerable free radical scavenging capacity [66,67,68,69,70,71]. The presence of these antioxidant compounds indicates that seaweeds can be good sources of polyphenols and could be utilized in food, feed, and pharmaceutical industries.

### 2.4. HPLC Quantitative Analysis

The quantitative analysis of targeted phenolic compounds was performed based on peak area computation using the calibration of corresponding standards and the result are presented as μg/g fresh weight of seaweeds (Table 4.). In total, seven polyphenols were targeted to quantify by HPLC-PDA, including six phenolic acids (gallic acid, caftaric acid, chlorogenic acid, caffeic acid, *p*-hydroxybenzoic acid and coumaric acid) and one flavonoid (catechin).

The most abundant targeted phenolic compound was *p*-hydroxybenzoic acid (Compound **5**), which was present in *Ulva* sp. with the concentration of 846.0 ± 0.02 μg/g_f.w._ The *p*-hydroxybenzoic acid content of eight green and red seaweeds in South Africa was previously reported as ranging from 0.51 ± 0.01 to 13.53 ± 0.03 μg/g dry weight (d.w.) [72], which was significantly lower than that of *Ulva* sp. in the present study. Gallic acid (Compound **1**), chlorogenic acid (Compound **2**) and caftaric acid (Compound **4**) were detected in *Centroceras* sp. with the concentration of 138.9 ± 0.02 μg/g_f.w._, 122.7 ± 0.01 μg/g_f.w._ and 19.7 ± 0.01 μg/g_f.w._, respectively. Coumaric acid (Compound **6**) was quantified in *Ulva* sp. with concentrations of 505.4 ± 0.03 μg/g_f.w_. Caffeic acid (Compound **3**) and catechin (Compound **7**) were present in *Caulerpa* sp. with a concentration of 612.9 ± 0.02 μg/g_f.w._ and 29.5 ± 0.03 μg/g_f.w._, respectively. Concentrations of gallic acid, chlorogenic acid and caffeic acid in brown seaweed *Himanthalia elongate* were also previously reported, being measured as 96.3 ± 3.12 μg/g_d.w._, 38.8 ± 1.94 μg/g_d.w._ and 44.4 ± 2.72 μg/g_d.w._, respectively [33]. About 10 marine-derived pharmaceutical drugs were approved by the Food and Drug Administration (FDA), and 30 candidates were in different stages of clinical trials for application in a number of disease areas [73]. The presence of these abundant polyphenols provide evidence for seaweeds as a good source of antioxidants for application in food and pharmaceutical industries, while further toxicity, pharmacological and clinical studies are needed. 

## 3. Materials and Methods 

### 3.1. Chemicals and Reagents

Unless otherwise stated, all chemicals used for extraction, characterization and antioxidant assays were analytical grade and purchased from Sigma-Aldrich (Castle Hill, NSW, Australia). Gallic acid, quercetin, catechin, ascorbic acid, 2,2-diphenyl-1-picrylhydrazyl (DPPH), 2,4,6-tripyridyl-s-triazine (TPTZ), aluminum chloride, iron (III) chloride, vanillin, potassium persulfate and 2,2’-azino-bis(3-ethylbenzothiazoline-6-sulphonic acid) (ABTS) were purchased from Sigma-Aldrich (Castle Hill, NSW, Australia). Sulfuric acid 98% was from RCI Labscan (Rongmuang, Thailand) and sodium carbonate anhydrous was from Chem-Supply Pty Ltd. (Adelaide, SA, Australia). Analytical-grade methanol, ethanol, hydrochloric acid, anhydrous sodium acetate and hydrated sodium acetate were from Fisher Chemical (Waltham, MA, USA). Acetic acid solution and acetonitrile, which comprised the mobile phases for HPLC and LC-MS, were from Sigma-Aldrich (St. Louis, MO, USA) and LiChrosolv (Darmstadt, Germany), respectively. The HPLC reference standards including gallic acid, caftaric acid, chlorogenic acid, caffeic acid, *p*-hydroxybenzoic acid, coumaric acid and catechin, were purchased from Sigma-Aldrich (St. Louis, MO, USA). Water was deionized to reach a resistivity of 18.2 MΩ/cm using a Millipore Milli-Q Gradient Water Purification System (Darmstadt, Germany) and was filtered through a 0.45 µm type Millipak^®^ Express 20 Filter (Milli-Q, Darmstadt, Germany).

### 3.2. Sample Preparation and Extraction of Polyphenols

Eight seaweeds which were identified as Chlorophyta (green; *Ulva* sp., *Caulerpa* sp. and *Codium* sp.), Rhodophyta (Red; *Dasya* sp., *Grateloupia* sp. and *Centroceras* sp.) and Ochrophyta (Brown; *Ecklonia* sp. and *Sargassum* sp.) were freshly collected from Brighton Beach in March 2019, VIC, Australia. Seaweeds were morphologically identified to the genus level. Classifications for Rhodophyta and Chlorophyta were verified using cytochrome c oxidase subunit I (COI-5P) and Elongation factor Tu 1-*Escherichia coli* (strain K12) tufA sequence data, respectively, following the protocol of Saunders and Kucera [74].

Extracts were prepared by modifying the previous studies [75,76], 2 g of each seaweed was grounded and mixed with 10 mL of 80% ethanol followed by homogenization using an Ultra-Turrax^®^ T25 homogenizer (Rawang, Selangor, Malaysia) at 10,000 rpm for 20 s. Then, incubation was carried out in a shaking incubator (ZWYR-240, Labwit, Ashwood, VIC, Australia) at 120 rpm at 4 °C for 16 h. Then, all the samples were centrifuged (Hettich Rotina 380R, Tuttlingen, Germany) at 10,000 rpm for 10 min. The supernatant was collected and stored at −20 °C for further analysis. For HPLC and LC-MS analysis, the extracts were filtered through a 0.45 μm syringe filter (Thermo Fisher Scientific Inc., Waltham, MA, USA).

### 3.3. Estimation of Polyphenols and Antioxidant Assays

For polyphenol estimation, TPC, TFC and TTC were measured, while for antioxidant potential, three different antioxidant assays, including DPPH, FRAP, and ABTS, were performed using the method of Feng, et al. [77]. The data were obtained by the Multiskan^®^ Go microplate photometer (Thermo Fisher Scientific, Waltham, MA, USA).

#### 3.3.1. Total Phenolic Content (TPC)

The total phenolic content of seaweed was determined using the Folin-Ciocalteu’s method [13] with some modifications. Twenty-five microliters of standards and samples (supernatant), 25 µL of 25% (*v/v*) folin reagent solution and 200 µL water were added to the wells in a 96-well plate (Corning Inc., Corning, NY, USA) and incubated at 25 °C for 5 min. Then, 25 µL of 10% (*w/w*) sodium carbonate was added and further incubated for 1 h at 25°C. The absorbance was measured at 765 nm against a blank using a Multiskan^®^ Go microplate photometer (Thermo Fisher Scientific, Waltham, MA, USA). The calibration curve was plotted using a gallic acid standard ranging from 0 to 200 µg/mL in ethanolic solution and the results were presented as microgram equivalents of gallic acid equivalents (GAE) per gram ± standard error (SE) on the basis of fresh weight (f.w.) (y = 0.0059x + 0.0593, R^2^ = 0.9996).

#### 3.3.2. Total Flavonoid Content (TFC)

The total flavonoid content was measured by aluminum chloride colorimetry according to Chan, Matanjun, Yasir and Tan [25], with some modifications. Methanolic quercetin standards and samples (80 µL) were added to the 96-well plate. Then, 80 µL of 2% (*w/v*) aluminum chloride (diluted with analytical grade ethanol) and 120 µL 50 g/L sodium acetate was added the wells in the plate followed by the incubation at 25 °C for 2.5 h in the dark. The calibration curve was plotted using quercetin standards ranging from 0 to 50 μg/mL and the results are presented as microgram equivalents of quercetin equivalents (QE)/g_f.w._ ± SE (y = 0.0195x + 0.0646, R^2^ = 0.999).

#### 3.3.3. Total Tannins Content (TTC)

Total tannin content was measured by modifying the method of Rebaya, et al. [78]. Sample/standard (25 µL of supernatant or standard), 150 µL 4% (*w/v*) methanolic vanillin solution and 25 µL 32% *(v/v*) sulfuric acid (diluted with methanol) were mixed in a 96-well plate and incubated at room temperature for 15 min. The absorbance was measured at 500 nm wavelength against a blank using the microplate reader. The calibration curve was plotted by catechin methanolic solution ranging from 0 to 1000 µg/mL and the results are presented as microgram equivalents of catechin (CE)/g_f.w._ ± SE (y = 0.0005x + 0.0578, R^2^ = 0.9854).

#### 3.3.4. 2,2-diphenyl-1-picrylhydrazyl (DPPH) Assay

DPPH radical scavenging activities of different extracts were determined based on Chan et al. [25] with some modifications. Quantities of 40 µL samples/standards and 260 µL of 0.1 mM methanolic DPPH were added to a 96-well plate. The reaction mixture was incubated for 30 min in the dark at room temperature, and the absorbance was measured under 517 nm wavelength against a blank. The standard curve was plotted by ascorbic acid aqueous solution ranging from 0 to 50 μg/mL and the results are expressed as the microgram equivalents of ascorbic acid (AAE)/g_f.w._ ± SE (y = −0.0089x + 0.5988, R^2^ = 0.9708). 

#### 3.3.5. Ferric Reducing Antioxidant Power (FRAP) Assay

The ferric reducing capabilities of the samples were measured using the FRAP method described by Matanjun, et al. [79], with slight modifications. The FRAP reagent was freshly prepared by mixing 300 mM acetate buffer, 10 mM TPTZ solution and 20 mM ferric chloride in the ratio of 10:1:1 (*v/v*). 20 µL samples/standards were added into the 96-well plate and mixed with 280 µL FRAP reagent. The mixture was incubated at 37 °C in the plate reader for 10 min before absorbance was measured at 593 nm. A standard curve was generated using ascorbic acid aqueous solution ranging from 0 to 50 µg/mL and the results are expressed as the microgram AAE/g_f.w._ ± SE (y = 0.009x + 0.403, R^2^ = 0.9819).

#### 3.3.6. 2,2′-Azino-bis-3-ethylbenzothiazoline-6-sulfonic Acid (ABTS) assay

The antioxidant activities of seaweeds were also measured by an ABTS assay according to Matanjun, Mohamed, Mustapha, Muhammad and Ming [79], with some modifications. ABTS^+^ was prepared by mixing 5 mL of 7 mM ABTS solution and 88 µL of 140 mM potassium persulfate solution, and the mixture was placed in the dark for 16 h to allow free radical generation. The stock solution was further diluted with 45 mL analytical-grade ethanol while the absorbance of the dye was fixed at approximately 0.7 at 734 nm. Quantities of 10 µL of sample/standards and 290 µL prepared dye solution were added into a 96-well plate followed by incubation at room temperature for 6 min and the absorbance was measured at 734 nm wavelength. The standard curve was plotted using ascorbic acid aqueous solution ranging from 0 to 200µ/mL and the results are expressed as the microgram AAE/g_f.w._ ± SE (y = -0.0042x + 0.6923, R^2^ = 0.9962).

### 3.4. LC-ESI-QTOF-MS/MS Characterization of Phenolic Compounds

LC-ESI-QTOF-MS/MS analysis was performed with an Agilent 1200 series HPLC (Agilent Technologies, Santa Clara, CA, USA) equipped with an Agilent 6520 Accurate-Mass Q-TOF LC-MS (Agilent Technologies, Santa Clara, CA, USA) via an electrospray ionization source (ESI). The separation was achieved by a Synergi Hydro-RP 80 Å, LC Column (250 mm × 4.6 mm, 4 µm) (Phenomenex, Lane Cove, NSW, Australia) at room temperature and the sample temperature was set at 10 °C. LC-MS/MS analysis were performed by modifying the method of Chao et al [66]. The mobile phase consisted of water/acetic acid (98:2, *v/v*; eluent A) and acetonitrile/acetic acid/ water (50:0.5:49.5, *v/v/v*; eluent B). The gradient profile was described as follows: 10–25% B (0–25 min), 25–35% B (25–35 min), 35–40% B (35–45 min), 40–55% B (45–75 min), 55–80% B (75–79 min), 80–90% B (79–82 min), 90–100% B (82–84 min), 100–10% B (84–87 min), isocratic 10% B (87–90 min). A volume of 6 µL was injected for each standard or sample and the flow rate was set at 0.8 mL/min. Nitrogen gas nebulization was set at 45 psi with a flow rate of 5L/min at 300 °C and the sheath gas was set at 11 L/min at 250 °C. The capillary and nozzle voltage were set at 3.5 kV and 500 V, respectively. A complete mass scan ranging from *m/z* 50 to 1300 was used, MS/MS analyses were carried out in automatic mode with collision energy (10, 15 and 30 eV) for fragmentation. Peak identification was performed in both positive and negative modes while the instrument control, data acquisition and processing were performed using MassHunter workstation software (Qualitative Analysis, version B.03.01) (Agilent Technologies, Santa Clara, CA, USA).

### 3.5. HPLC-PDA Quantitative Analysis of Individual Phenolic Compounds

The quantitative measurement of individual phenolic compounds present in seaweed samples was performed with an Agilent 1200 HPLC equipped with a photodiode array (PDA) detector by adopting the protocol of Peng et al. [68]. The same column and conditions were used as described above in LC-ESI-QTOF-MS/MS, except for a sample injection volume of 20 µL. The compositions of extracts were detected under λ 280 nm, 320 nm, and 370 nm by PDA detector simultaneously with 1.25 scan/s (peak width = 0.2 min) spectral acquisition rate. The targeted phenolic compounds were quantified based on linear regression of external standards peak area against concentration. Data acquisition and analysis were performed by MassHunter workstation software—version B.03.01 (Agilent Technologies, Santa Clara, CA, USA).

### 3.6. Statistical Analysis

All analyses were performed in triplicates and the results are presented as mean ± standard error (n = 3). Data were analyzed using Tukey’s one-way analysis of variance (ANOVA) by Minitab^®^ 19 for windows (Minitab, NSW, Australia). A significant difference was considered at the level of *p* ≤ 0.05 using Tukey’s HSD test.

## 4. Conclusions

Brown seaweed species showed significantly higher polyphenolic content and potential antioxidant capacity than green and red seaweeds. The antioxidant properties varied across different species. Application of LC-ESI-QTOF-MS/MS enabled the isolation and identification of 54 phenolic compounds present in seaweeds. Quantitative analysis of targeted compounds was achieved by calibration of standards using HPLC-PDA. Seven targeted compounds were quantified in seaweeds, with *p*-hydroxybenzoic acid being the most abundant. This is the first report that applied different antioxidant assays to estimate the antioxidant potential and applied LC-MS technique to isolate and characterize the polyphenols in some abundant Australian seaweed species. The presence of the various polyphenols with antioxidant potential was identified. Further toxicity, pharmacological and clinical studies should be explored before the application of these Australian seaweeds as ingredients in food, nutraceuticals and pharmaceutical products.

## Figures and Tables

**Table 1:** Phenolic content estimated in the seaweeds investigated in this study.

Samples.	TPC (μg GAE/g)	TFC (μg QE/g)	TTC (μg CE/g)
**Green seaweeds**			
*Ulva* sp.	14.80 ± 0.54 ^d^	9.80 ± 1.96 ^de^	-
*Caulerpa* sp.	4.30 ± 0.45 ^d^	0.73 ± 0.08 ^f^	3.31 ± 7.02 ^b^
*Codium* sp.	2.29 ± 0.26 ^d^	1.11 ± 0.63 ^f^	-
Red seaweeds *			
*Dasya* sp.	260.15 ± 2.25 ^c^	29.96 ± 0.48 ^c^	24.90 ± 3.46 ^b^
*Grateloupia* sp.	524.56 ± 0.46 ^b^	54.43 ± 0.74 ^a^	-
*Centroceras* sp.	49.31 ± 2.17 ^d^	42.55 ± 0.52 ^b^	4.45 ± 4.37 ^b^
Brown seaweeds *			
*Ecklonia* sp.	1044.36 ± 2.54 ^a^	13.87 ± 1.18 ^d^	166.87 ± 23.24 ^a^
*Sargassum* sp.	22.27 ± 0.15 ^d^	3.88 ± 0.27 ^ef^	5.62 ± 0.01 ^b^

The data are shown as mean ± standard error (n = 3); the superscript letters (a–f), indicate the means within a column with significant difference (*p* < 0.05) using a one-way analysis of variance (ANOVA) and Tukey’s test. Data of seaweed is reported on a fresh weight basis. *: total polyphenol content of brown seaweeds was significantly higher than green and red seaweeds; total flavonoid content of red seaweeds was significantly higher than green and brown seaweeds (*p* < 0.05). The phenolic content, as measured by total phenolic content (TPC), total flavonoid content (TFC), total tannin contents (TTC). GAE stands for gallic acid equivalents, QE stands for quercetin equivalents and CE stands for catechin equivalents.

**Table 2:** Antioxidant activities detected in the seaweeds investigated in this study.

Samples	ABTS (μg AAE/g)	DPPH (μg AAE/g)	FRAP (μg AAE/g)
**Green seaweeds**			
*Ulva* sp.	14.24 ± 0.93 ^d^	-	4.10 ± 1.45 ^bc^
*Caulerpa* sp.	20.93 ± 2.62 ^d^	-	0.53 ± 0.05 ^c^
*Codium* sp.	10.05 ± 6.65 ^d^	-	1.07 ± 0.62 ^c^
**Red seaweeds**			
*Dasya* sp.	179.63 ± 9.3 ^c^	12.71 ± 0.83 ^b^	27.39 ± 1.47 ^bc^
*Grateloupia* sp.	243.06 ± 3.78 ^b^	19.12 ± 0.64 ^b^	35.05 ± 1.54 ^b^
*Centroceras* sp.	27.91 ± 3.79 ^d^	6.30 ± 0.73 ^b^	1.86 ± 1.15 ^c^
Brown seaweeds *			
*Ecklonia* sp.	957.85 ± 0.36 ^a^	510.32 ± 3.38 ^a^	170.03 ± 2.04 ^a^
*Sargassum* sp.	42.62 ± 3.09 ^d^	13.71 ± 5.67 ^b^	4.76 ± 0.48 ^bc^

The data are shown as mean ± standard error (n = 3); the superscript letters (a–d), indicate the means within a column with significant difference (*p* < 0.05) using a one-way analysis of variance (ANOVA) and Tukey’s test. Data of seaweed is reported on a fresh weight basis. *: Antioxidant capacities of brown seaweeds are significantly higher than that of green and red seaweeds (*p* < 0.05). DPPH stands for 2,2-diphenyl-1-picrylhydrazyl, ABTS stands for 2,2′-azino-bis-3-ethylbenzothiazoline-6-sulfonic acid and FRAP stands for ferric reducing antioxidant power assay. AAE stands for ascorbic acid equivalents.

**Table 3:** Characterization of phenolic compounds in seaweeds by using LC-ESI-QTOF-MS/MS.

No.	Proposed Compounds	Molecular Formula	RT (min)	Ionization (ESI^+^/ESI^-^)	Molecular Weight	Theoretical (*m/z*)	Observed (*m/z*)	Mass Error (ppm)	MS/MS Product Ions	Seaweeds
**Phenolic acid**										
**Hydroxybenzoic acids**										
1	Vanillic acid 4-sulfate	C_8_H_8_O_7_S	9.112	[M − H]^−^	247.9991	246.9918	246.9925	2.83	217, 203, 167	* *Sargassum* sp., *Centroceras* sp., *Ulva* sp.
2	Gallic acid	C_7_H_6_O_5_	9.885	** [M − H]^−^	170.0215	169.0142	169.0138	−2.37	125	*Centroceras* sp.
3	4-Hydroxybenzoic acid 4-*O*-glucoside	C_13_H_16_O_8_	11.515	[M − H]^−^	300.0845	299.0772	299.0778	2.01	255, 137	*Sargassum* sp.
4	Protocatechuic acid 4-*O*-glucoside	C_13_H_16_O_9_	13.546	** [M − H]^−^	316.0794	315.0721	315.0719	−0.63	153	* *Centroceras* sp., *Grateloupia* sp.
5	*p*-Hydroxybenzoic acid	C_7_H_6_O_3_	32.906	[M − H]^−^	138.0317	137.0244	137.0240	−2.91	93	* *Ulva* sp., *Caulerpa* sp., *Centroceras* sp.
6	Ellagic acid glucoside	C_20_H_16_O_13_	38.451	[M − H]^−^	464.0591	463.0518	463.0518	0.01	301	*Ecklonia* sp.
**Hydroxycinnamic acids**										
7	3-Sinapoylquinic acid	C_18_H_22_O_10_	7.005	** [M − H]^−^	398.1213	397.1140	397.1144	1.01	223, 179	* *Centroceras* sp., *Ecklonia* sp.
8	Cinnamoyl glucose	C_15_H_18_O_7_	8.861	** [M − H]^−^	310.1053	309.098	309.0992	3.88	147, 131, 103	* *Codium* sp., *Ulva* sp.
9	Caffeoyl glucose	C_15_H_18_O_9_	10.983	** [M − H]^−^	342.0951	341.0878	341.0882	1.17	179, 161	* *Ecklonia* sp., *Centroceras* sp.
10	Caffeic acid 3-*O*-glucuronide	C_15_H_16_O_10_	14.259	** [M − H]^−^	356.0743	355.0670	355.0671	0.28	179	*Caulerpa* sp.
11	Chlorogenic acid	C_16_H_18_O_9_	15.004	** [M − H]^−^	354.0951	353.0878	353.0862	−4.53	253, 190, 144	* *Centroceras* sp., *Caulerpa* sp.
12	Caffeic acid	C_9_H_8_O_4_	18.274	[M − H]^−^	180.0423	179.0350	179.0350	0.01	151, 143, 133	*Caulerpa* sp.
13	Caffeic acid 4-sulfate	C_9_H_8_O_7_S	18.291	[M − H]^−^	259.9991	258.9918	258.9929	4.25	215, 179, 135	*Caulerpa* sp.
14	Caffeoyl tartaric acid	C_13_H_12_O_9_	24.061	** [M − H]^−^	312.0481	311.0408	311.0403	−1.61	161	* *Grateloupia* sp., *Centroceras* sp.
15	Isoferulic acid 3-sulfate	C_10_H_10_O_7_S	24.520	** [M − H]^−^	274.0147	273.0074	273.0086	4.4	193, 149	*Caulerpa* sp.
16	Sinapic acid	C_11_H_12_O_5_	25.852	** [M − H]^−^	224.0685	223.0612	223.0621	4.03	205, 179, 163	* *Ulva* sp., *Caulerpa* sp., *Grateloupia* sp.
17	Ferulic acid	C_10_H_10_O_4_	32.604	[M − H]^−^	194.0579	193.0506	193.0513	3.63	178, 149, 134	*Caulerpa* sp.
18	Coumaric acid	C_9_H_8_O_3_	33.797	** [M − H]^−^	164.0473	163.0400	163.0406	3.68	119	* *Ulva* sp., *Ecklonia* sp.
19	Sinapine	C_16_H_24_NO_5_	88.066	[M + H]^+^	310.1652	310.1654	310.1646	−2.58	251, 207, 175	*Codium* sp.
**Hydroxyphenylpentanoic acids**										
20	5-(3′,5′-dihydroxyphenyl)-γ-valerolactone 3-*O*-glucuronide	C_17_H_20_O_10_	14.855	** [M − H]^−^	384.1056	383.0983	383.1001	4.70	221, 206, 191	* *Ecklonia* sp., *Codium* sp.
21	5-(3′,4′-dihydroxyphenyl)-valeric acid	C_11_H_14_O_4_	51.563	** [M − H]^−^	210.0892	209.0819	209.0821	0.96	165, 150	*Caulerpa* sp.
**Hydroxyphenylacetic acids**										
22	2-Hydroxy-2-phenylacetic acid	C_8_H_8_O_3_	6.18	** [M + H]^+^	152.0473	153.0546	153.055	2.61	125	* *Centroceras* sp., *Caulerpa* sp., *Sargassum* sp.
**Flavonoids**										
**Anthocyanins**										
23	Delphinidin 3-*O*-sambubioside	C_26_H_29_O_16_	9.327	[M + H]^+^	597.1464	597.1456	597.1473	2.85	303, 257, 229	*Grateloupia* sp.
24	Isopeonidin 3-*O*-arabinoside	C_21_H_21_O_10_	41.658	[M + H]^+^	433.1134	433.1135	433.1136	0.23	271, 253, 243	*Centroceras* sp.
25	Malvidin 3-*O*-glucoside	C_23_H_25_O_12_	54.152	[M + H]^+^	493.1343	493.1346	493.1343	−0.61	331	*Centroceras* sp.
**Flavanols**										
26	Gallocatechin	C_15_H_14_O_7_	7.604	** [M − H]^−^	306.0740	305.0667	305.0668	0.33	261, 219	* *Caulerpa* sp., *Ulva* sp., *Dasya* sp., *Ecklonia* sp., *Sargassum* sp.
27	3′-*O*-Methylcatechin	C_16_H_16_O_6_	17.857	** [M − H]^−^	304.0947	303.0874	303.0886	3.96	271, 163	*Grateloupia* sp.
28	Catechin (isomer)	C_15_H_14_O_6_	45.118	[M − H]^−^	290.0790	289.0717	289.0731	4.84	245, 205, 179	*Caulerpa* sp.
**Flavonols**										
29	Quercetin 3-*O*-(6”-malonyl-glucoside)	C_24_H_22_O_15_	9.902	[M − H]^−^	550.0959	549.0886	549.0887	0.18	463, 301, 161	* *Centroceras* sp., *Caulerpa* sp.
30	5,3′,4′-Trihydroxy-3-methoxy-6:7-methylenedioxyflavone 4’-*O*-glucuronide	C_23_H_20_O_14_	33.878	[M − H]^−^	520.0853	519.0780	519.0779	−0.19	343	*Ecklonia* sp.
31	3,7-Dimethylquercetin	C_17_H_14_O_7_	80.642	[M − H]^−^	330.0740	329.0667	329.0674	2.13	314, 299, 271	*Centroceras* sp.
**Flavones**										
32	Rhoifolin	C_27_H_30_O_14_	44.036	** [M − H]^−^	578.1636	577.1563	577.1588	4.33	413, 269	*Centroceras* sp.
**Isoflavonoids**										
33	Sativanone	C_17_H_16_O_5_	4.240	[M − H]^−^	300.0998	299.0925	299.0918	−2.34	284, 269, 225	*Ecklonia* sp.
34	Glycitein 7-*O*-glucuronide	C_22_H_20_O_11_	4.454	** [M − H]^−^	460.1006	459.0933	459.0923	−2.18	283, 268, 117	*Centroceras* sp.
35	3′,4′,5,7-Tetrahydroxyisoflavanone	C_15_H_12_O_6_	4.640	** [M − H]^−^	288.0634	287.0561	287.0556	−1.74	269, 259	*Caulerpa sp., *Grateloupia* sp., *Centroceras* sp.
36	3’-*O*-Methylequol	C_16_H_16_O_4_	4.803	** [M − H]^−^	272.1049	271.0976	271.0972	−1.48	147, 123, 121	*Ecklonia sp., *Grateloupia* sp.
37	Dalbergin	C_16_H_12_O_4_	9.344	** [M − H]^−^	268.0736	267.0663	267.0666	1.12	252, 224, 180	* *Grateloupia* sp., *Centroceras* sp.
38	Dihydrobiochanin A	C_16_H_14_O_5_	80.715	** [M − H]^−^	286.0841	285.0768	285.0771	1.05	270	* *Codium* sp., *Centroceras* sp.
39	3′-Hydroxydaidzein	C_15_H_10_O_5_	86.956	[M − H]^−^	270.0528	269.0455	269.0457	0.74	151, 117, 107	* *Grateloupia* sp., *Centroceras* sp., *Caulerpa* sp., *Ecklonia* sp.
**Other polyphenols**										
**Hydroxybenzaldehydes**										
40	*p*-Hydroxybenzaldehyde	C_7_H_6_O_2_	15.921	[M − H]^−^	122.0368	121.0295	121.0295	0.01	92, 77	* *Dasya* sp., *Ecklonia* sp., *Codium* sp.
**Hydroxycoumarins**										
41	Urolithin A	C_13_H_8_O_4_	4.64	[M − H]^−^	228.0423	227.0350	227.0341	−3.96	198, 182	*Grateloupia* sp.
42	Scopoletin	C_10_H_8_O_4_	84.705	** [M − H]^−^	192.0423	191.0350	191.0352	1.05	176, 147	* *Codium* sp., *Grateloupia* sp., *Sargassum* sp.
**Phenolic terpenes**										
43	Rosmanol	C_20_H_26_O_5_	24.965	[M + H]^+^	346.1780	347.1853	347.1843	−2.88	301, 231	* *Dasya* sp., *Ulva* sp., *Grateloupia* sp., *Ecklonia* sp., *Codium* sp.
44	Carnosol	C_20_H_26_O_4_	85.931	** [M − H]^−^	330.1831	329.1758	329.1747	−3.34	287, 286, 285	* *Codium* sp., *Caulerpa* sp.
45	Carnosic acid	C_20_H_28_O_4_	86.958	** [M − H]^−^	332.1988	331.1915	331.1912	−0.91	287, 269	* *Ecklonia* sp., *Dasya* sp., *Codium* sp., *Sargassum* sp.
**Tyrosols**										
46	Hydroxytyrosol 4-*O*-glucoside	C_14_H_20_O_8_	36.653	** [M − H]^−^	316.1158	315.1085	315.1091	1.90	153, 123	* *Centroceras* sp., *Dasya* sp., *Grateloupia* sp., *Sargassum* sp.
47	3,4-DHPEA-EDA	C_17_H_20_O_6_	87.423	[M − H]^−^	320.1260	319.1187	319.1200	4.07	301, 275, 195	*Caulerpa* sp.
**Other polyphenols**										
48	3,4-Dihydroxyphenylglycol	C_8_H_10_O_4_	7.005	[M − H]^−^	170.0579	169.0506	169.0503	−1.77	141, 139, 123	*Centroceras* sp.
49	Phloroglucinol	C_6_H_6_O_3_	14.793	[M − H]^−^	126.0317	125.0244	125.0242	−1.59	97	* *Ecklonia* sp., *Sargassum* sp.
50	Isopropyl 3-(3,4-dihydroxyphenyl)-2-hydroxypropanoate	C_12_H_16_O_5_	24.882	** [M − H]^−^	240.0998	239.0925	239.0919	−2.51	195, 155, 99	*Dasya* sp.
**Lignans**										
**Lignan derivatives**										
51	2′-Hydroxyenterolactone	C_18_H_18_O_5_	7.781	[M − H]^−^	314.1154	313.1081	313.1082	0.32	295, 283	*Grateloupia* sp.
52	Arctigenin	C_21_H_24_O_6_	8.131	** [M − H]^−^	372.1573	371.1500	371.1509	2.42	356, 312, 295	* *Centroceras* sp., *Sargassum* sp.
53	Dimethylmatairesinol	C_22_H_26_O_6_	83.663	[M + H]^+^	386.1729	387.1802	387.1805	0.77	372, 369, 357, 329	* *Caulerpa* sp., *Dasya* sp.
54	Deoxyschisandrin	C_24_H_32_O_6_	85.152	** [M + H]^+^	416.2199	417.2272	417.2286	3.36	402, 347, 316, 301	* *Ecklonia* sp., *Codium* sp., *Sargassum* sp.

* Compound was detected in more than one seaweed samples, data presented in this table are from asterisk sample. ** Compounds were detected in both negative [M − H]^−^ and positive [M + H]^+^ mode of ionization while only single mode data was presented. RT = stands for “retention time”.

**Table 4:** Quantification of targeted phenolic compounds by high-performance liquid chromatography (HPLC) in seaweeds.

No.	Compound Name	Structure Formula	RT (min)	Concentration (μg/g_f.w._)	Seaweed Samples
1	Gallic acid	C_7_H_6_O_5_	9.685	138.887 ± 0.02	*Centroceras* sp.
2	Chlorogenic acid	C_16_H_18_O_9_	15.004	122.706 ± 0.01	*Centroceras* sp.
3	Caffeic acid	C_9_H_8_O_4_	18.274	612.824 ± 0.02	*Caulerpa* sp.
4	Caftaric acid	C_13_H_12_O_9_	24.532	19.667 ± 0.01	*Centroceras* sp.
5	*p*-hydroxybenzoic acid	C_7_H_6_O_3_	32.906	846.083 ± 0.02	*Ulva* sp.
6	Coumaric acid	C_9_H_8_O_3_	33.797	505.387 ± 0.03	*Ulva* sp.
7	Catechin	C_15_H_14_O_6_	64.081	29.469 ± 0.03	*Caulerpa* sp.

RT = stands for “retention time”.

## Supplementary Materials

The following are available online at https://www.mdpi.com/1660-3397/18/6/331/s1, Table S1: Characterization of phenolic compounds in seaweeds by using LC-ESI-QTOF-MS/MS. Figure S1: Base peak chromatogram (BPC) for characterization of phenolic compounds of seaweeds. Figure S2. Extracted ion chromatogram and mass spectrum of “Vanillic acid 4-sulfate” detected in three different seaweeds.

## References

[1] Ferdouse F., Holdt S.L., Smith R., Murua P., Yang Z. (2018). The Global Status of Seaweed Production, Trade and Utilization.

[2] Wyrepkowski C.C., Costa D.L., Sinhorin A.P., Vilegas W., De Grandis R.A., Resende F.A., Varanda E.A., dos Santos L.C. (2014). Characterization and quantification of the compounds of the ethanolic extract from caesalpinia ferrea stem bark and evaluation of their mutagenic activity. Molecules.

[3] Mouritsen O.G., Mouritsen J.D., Johansen M. (2013). Seaweeds: Edible, Available & Sustainable.

[4] Fleurence J., Fleurence J., Levine I. (2016). Chapter 5–Seaweeds as food. Seaweed in Health and Disease Prevention.

[5] Menon V.V., Lele S.S. (2015). Nutraceuticals and bioactive compounds from seafood processing waste. Springer Handbook of Marine Biotechnology.

[6] Suleria H.A., Masci P., Gobe G., Osborne S. (2016). Current and potential uses of bioactive molecules from marine processing waste. J. Sci. Food Agric..

[7] Maqsood S., Benjakul S., Shahidi F. (2013). Emerging role of phenolic compounds as natural food additives in fish and fish products. Crit. Rev. Food Sci. Nutr..

[8] Manach C., Scalbert A., Morand C., Remesy C., Jimenez L. (2004). Polyphenols: Food sources and bioavailability. Am. J. Clin. Nutr..

[9] Rajauria G., Foley B., Abu-Ghannam N. (2016). Identification and characterization of phenolic antioxidant compounds from brown irish seaweed himanthalia elongata using lc-dad-esi-ms/ms. Innov. Food Sci. Emerg. Technol..

[10] Valko M., Leibfritz D., Moncol J., Cronin M.T., Mazur M., Telser J. (2007). Free radicals and antioxidants in normal physiological functions and human disease. Int. J. Biochem. Cell Biol..

[11] Airanthi M.K., Hosokawa M., Miyashita K. (2011). Comparative antioxidant activity of edible japanese brown seaweeds. J. Food. Sci..

[12] Namvar F., Mohamad R., Baharara J., Zafar-Balanejad S., Fargahi F., Rahman H.S. (2013). Antioxidant, antiproliferative, and antiangiogenesis effects of polyphenol-rich seaweed (sargassum muticum). BioMed Res. Int..

[13] Lopez A., Rico M., Rivero A., de Tangil M.S. (2011). The effects of solvents on the phenolic contents and antioxidant activity of stypocaulon scoparium algae extracts. Food Chem..

[14] Leopoldini M., Russo N., Toscano M. (2011). The molecular basis of working mechanism of natural polyphenolic antioxidants. Food Chem..

[15] Kelman D., Posner E.K., McDermid K.J., Tabandera N.K., Wright P.R., Wright A.D. (2012). Antioxidant activity of hawaiian marine algae. Mar. Drugs.

[16] Kulawik P., Ozogul F., Glew R., Ozogul Y. (2013). Significance of antioxidants for seafood safety and human health. J. Agric. Food Chem..

[17] Kalita P., Tapan B.K., Pal T.K., Kalita R. (2013). Estimation of total flavonoids content (tfc) and anti oxidant activities of methanolic whole plant extract of biophytum sensitivum linn. JDDT.

[18] Lopes G., Barbosa M., Vallejo F., Gil-Izquierdo A., Andrade P.B., Valentao P., Pereira D.M., Ferreres F. (2018). Profiling phlorotannins from fucus spp. Of the northern portuguese coastline: Chemical approach by hplc-dad-esi/msn and uplc-esi-qtof/ms. Algal Res..

[19] Liu B., Kongstad K.T., Wiese S., Jäger A.K., Staerk D. (2016). Edible seaweed as future functional food: Identification of α-glucosidase inhibitors by combined use of high-resolution α-glucosidase inhibition profiling and hplc–hrms–spe–nmr. J. Food Chem..

[20] García-Casal M.N., Ramirez J., Leets I., Pereira A.C., Quiroga M.F. (2008). Antioxidant capacity, polyphenol content and iron bioavailability from algae (ulva sp., sargassum sp. And porphyra sp.) in human subjects. J. Food Chem..

[21] Sabeena Farvin K.H., Jacobsen C. (2013). Phenolic compounds and antioxidant activities of selected species of seaweeds from danish coast. Food Chem..

[22] Mekinic I.G., Skroza D., Simat V., Hamed I., Cagalj M., Perkovic Z.P. (2019). Phenolic content of brown algae (pheophyceae) species: Extraction, identification, and quantification. Biomolecules.

[23] Ford L., Theodoridou K., Sheldrake G.N., Walsh P.J. (2019). A critical review of analytical methods used for the chemical characterisation and quantification of phlorotannin compounds in brown seaweeds. Phytochem. Anal..

[24] Cox S., Abu-Ghannam N., Gupta S. (2010). An assessment of the antioxidant and antimicrobial activity of six species of edible irish seaweeds. J. Food Chem..

[25] Chan P.T., Matanjun P., Yasir S.M., Tan T.S. (2015). Antioxidant activities and polyphenolics of various solvent extracts of red seaweed, gracilaria changii. J. Food Chem..

[26] Wang T., Jonsdottir R., Ólafsdóttir G. (2009). Total phenolic compounds, radical scavenging and metal chelation of extracts from icelandic seaweeds. J. Food Chem..

[27] Pinteus S., Silva J., Alves C., Horta A., Fino N., Rodrigues A.I., Mendes S., Pedrosa R. (2017). Cytoprotective effect of seaweeds with high antioxidant activity from the peniche coast (portugal). Food Chem..

[28] Kim A.R., Shin T.S., Lee M.S., Park J.Y., Park K.E., Yoon N.Y., Kim J.S., Choi J.S., Jang B.C., Byun D.S. (2009). Isolation and identification of phlorotannins from ecklonia stolonifera with antioxidant and anti-inflammatory properties. J. Agric. Food Chem..

[29] Schaich K.M., Tian X., Xie J. (2015). Hurdles and pitfalls in measuring antioxidant efficacy: A critical evaluation of abts, dpph, and orac assays. J. Funct. Foods.

[30] Sachindra N.M., Airanthi M.K., Hosokawa M., Miyashita K. (2010). Radical scavenging and singlet oxygen quenching activity of extracts from indian seaweeds. J. Food Sci. Technol..

[31] Ramon-Goncalves M., Gomez-Mejia E., Rosales-Conrado N., Leon-Gonzalez M.E., Madrid Y. (2019). Extraction, identification and quantification of polyphenols from spent coffee grounds by chromatographic methods and chemometric analyses. Waste Manag..

[32] Escobar-Avello D., Lozano-Castellon J., Mardones C., Perez A.J., Saez V., Riquelme S., von Baer D., Vallverdu-Queralt A. (2019). Phenolic profile of grape canes: Novel compounds identified by lc-esi-ltq-orbitrap-ms. Molecules.

[33] Rajauria G. (2018). Optimization and validation of reverse phase hplc method for qualitative and quantitative assessment of polyphenols in seaweed. J. Pharm. Biomed. Anal..

[34] Dinh T.V., Saravana P.S., Woo H.C., Chun B.S. (2018). Ionic liquid-assisted subcritical water enhances the extraction of phenolics from brown seaweed and its antioxidant activity. Sep. Purif. Technol..

[35] Lin L.Z., Harnly J.M. (2008). Identification of hydroxycinnamoylquinic acids of arnica flowers and burdock roots using a standardized lc-dad-esi/ms profiling method. J. Agric. Food Chem..

[36] Yang D.-z., Sun G., Zhang A., Fu S., Liu J.-h. (2015). Screening and analyzing the potential bioactive components from rhubarb, using a multivariate data processing approach and ultra-high performance liquid chromatography coupled with time-of-flight mass spectrometry. Anal. Methods.

[37] Wang X., Liu J., Zhang A., Sun H., Zhang Y., Wang X. (2017). Chapter 23–Systematic characterization of the absorbed components of acanthopanax senticosus stem. Serum Pharmacochemistry of Traditional Chinese Medicine.

[38] Al-Ayed A.S. (2015). Integrated mass spectrometry approach to screening of phenolic molecules in hyphaene thebiaca fruits with their antiradical activity by thin-layer chromatography. Indian J. Chem. Technol..

[39] De Oliveira D.N., de Bona Sartor S., Damário N., Gollücke A.P., Catharino R.R. (2014). Antioxidant activity of grape products and characterization of components by electrospray ionization mass spectrometry. J. Food Meas. Charact..

[40] Lin H.Q., Zhu H.L., Tan J., Wang H., Wang Z.Y., Li P.Y., Zhao C.F., Liu J.P. (2019). Comparative analysis of chemical constituents of moringa oleifera leaves from china and india by ultra-performance liquid chromatography coupled with quadrupole-time-of-flight mass spectrometry. Molecules.

[41] Tong T., Li J., Ko D.-O., Kim B.-S., Zhang C., Ham K.-S., Kang S.-G. (2014). In vitro antioxidant potential and inhibitory effect of seaweed on enzymes relevant for hyperglycemia. Food Sci. Biotechnol..

[42] Geng C.A., Chen H., Chen X.L., Zhang X.M., Lei L.G., Chen J.J. (2014). Rapid characterization of chemical constituents in saniculiphyllum guangxiense by ultra fast liquid chromatography with diode array detection and electrospray ionization tandem mass spectrometry. Int. J. Mass Spectrom..

[43] Wang J., Jia Z., Zhang Z., Wang Y., Liu X., Wang L., Lin R. (2017). Analysis of chemical constituents of melastoma dodecandrum lour. By uplc-esi-q-exactive focus-ms/ms. Molecules.

[44] Agregán R., Munekata P.E., Franco D., Dominguez R., Carballo J., Lorenzo J.M. (2017). Phenolic compounds from three brown seaweed species using lc-dad–esi-ms/ms. Food Res. Int..

[45] Olate-Gallegos C., Barriga A., Vergara C., Fredes C., Garcia P., Gimenez B., Robert P. (2019). Identification of polyphenols from chilean brown seaweeds extracts by lc-dad-esi-ms/ms. J. Aquat. Food Prod. Technol..

[46] Rajauria G. (2019). In-vitro antioxidant properties of lipophilic antioxidant compounds from 3 brown seaweed. Antioxidants.

[47] Reed K.A. (2009). Identification of Phenolic Compounds from Peanut Skin Using hplc-ms^n^.

[48] Zhang L., Tu Z.-C., Wang H., Fu Z.-F., Wen Q.-H., Chang H.-X., Huang X.-Q. (2015). Comparison of different methods for extracting polyphenols from ipomoea batatas leaves, and identification of antioxidant constituents by hplc-qtoe-ms2. Food Res. Int..

[49] Zeng X., Su W., Zheng Y., Liu H., Li P., Zhang W., Liang Y., Bai Y., Peng W., Yao H. (2018). Uflc-q-tof-ms/ms-based screening and identification of flavonoids and derived metabolites in human urine after oral administration of exocarpium citri grandis extract. Molecules.

[50] Zhao X., Zhang S., Liu D., Yang M., Wei J. (2020). Analysis of flavonoids in dalbergia odorifera by ultra-performance liquid chromatography with tandem mass spectrometry. Molecules.

[51] Tanna B., Brahmbhatt H.R., Mishra A. (2019). Phenolic, flavonoid, and amino acid compositions reveal that selected tropical seaweeds have the potential to be functional food ingredients. J. Food Process. Preserv..

[52] Wang Y., Vorsa N., Harrington P.d.B., Chen P. (2018). Nontargeted metabolomic study on variation of phenolics in different cranberry cultivars using uplc-im-hrms. J. Agric. Food Chem..

[53] Wu S.-H., Li H.-B., Li G.-L., Lv N., Qi Y.-J. (2020). Metabolite identification of gut microflora-cassia seed interactions using uplc-qtof/ms. Exp. Ther. Med..

[54] Zeng Y., Lu Y., Chen Z., Tan J., Bai J., Li P., Wang Z., Du S. (2018). Rapid characterization of components in bolbostemma paniculatum by uplc/ltq-orbitrap msn analysis and multivariate statistical analysis for herb discrimination. Molecules.

[55] Jesionek W., Majer-Dziedzic B., Horvath G., Moricz A.M., Choma I.M. (2017). Screening of antibacterial compounds in salvia officinalis l. Tincture using thin-layer chromatography-direct bioautography and liquid chromatography-tandem mass spectrometry techniques. JPC J. Planar Chromatogr. Mod. TLC.

[56] Pacifico S., Piccolella S., Lettieri A., Nocera P., Bollino F., Catauro M. (2017). A metabolic profiling approach to an italian-sage leaf extract (soa541) defines its antioxidant and anti-acetylcholinesterase properties. J. Funct. Foods.

[57] Hermund D., Jacobsen C., Chronakis I.S., Pelayo A., Yu S., Busolo M., Lagaron J.M., Jónsdóttir R., Kristinsson H.G., Akoh C.C. (2019). Stabilization of fish oil-loaded electrosprayed capsules with seaweed and commercial natural antioxidants: Effect on the oxidative stability of capsule-enriched mayonnaise. Eur. J. Lipd Sci. Technol..

[58] Di Maio I., Esposto S., Taticchi A., Selvaggini R., Veneziani G., Urbani S., Servili M. (2013). Characterization of 3,4-dhpea-eda oxidation products in virgin olive oil by high performance liquid chromatography coupled with mass spectrometry. Food Chem..

[59] Gomez-Alonso S., Salvador M.D., Fregapane G. (2002). Phenolic compounds profile of cornicabra virgin olive oil. J. Agric. Food Chem..

[60] Salama M., El-Hawary S., Mousa O., El- Askary N., Esmat A. (2015). In vivo tnf-α and il-1β inhibitory activity of phenolics isolated from trachelospermum jasminoides (lindl.) lem. Med. Plants Res..

[61] Yang S., Shan L., Luo H., Sheng X., Du J., Li Y. (2017). Rapid classification and identification of chemical components of schisandra chinensis by uplc-q-tof/ms combined with data post-processing. Molecules.

[62] Dhargalkar V. (2015). Uses of seaweeds in the indian diet for sustenance and well-being. Sci. Cult..

[63] Rodriguez-Garcia C., Sanchez-Quesada C., Toledo E., Delgado-Rodriguez M., Gaforio J.J. (2019). Naturally lignan-rich foods: A dietary tool for health promotion?. Molecules.

[64] Peterson J., Dwyer J., Adlercreutz H., Scalbert A., Jacques P., McCullough M.L. (2010). Dietary lignans: Physiology and potential for cardiovascular disease risk reduction. Nutr. Rev..

[65] Kershaw J., Kim K.H. (2017). The therapeutic potential of piceatannol, a natural stilbene, in metabolic diseases: A review. J. Med. Food.

[66] Ma C., Dunshea F.R., Suleria H.A.R. (2019). Lc-esi-qtof/ms characterization of phenolic compounds in palm fruits (jelly and fishtail palm) and their potential antioxidant activities. Antioxidants.

[67] Tang J., Dunshea F.R., Suleria H.A.R. (2020). Lc-esi-qtof/ms characterization of phenolic compounds from medicinal plants (hops and juniper berries) and their antioxidant activity. Foods.

[68] Peng D., Zahid H.F., Ajlouni S., Dunshea F.R., Suleria H.A.R. (2019). Lc-esi-qtof/ms profiling of australian mango peel by-product polyphenols and their potential antioxidant activities. Processes.

[69] Yang P., Xu F., Li H.F., Wang Y., Li F.C., Shang M.Y., Liu G.X., Wang X., Cai S.Q. (2016). Detection of 191 taxifolin metabolites and their distribution in rats using hplc-esi-it-tof-msn. Molecules.

[70] Oszmiański J., Kolniak-Ostek J., Wojdyło A. (2013). Application of ultra performance liquid chromatography-photodiode detector-quadrupole/time of flight-mass spectrometry (uplc-pda-q/tof-ms) method for the characterization of phenolic compounds of lepidium sativum l. Sprouts. Eur. Food Res. Technol..

[71] Kim I., Lee J. (2020). Variations in anthocyanin profiles and antioxidant activity of 12 genotypes of mulberry (morus spp.) fruits and their changes during processing. Antioxidants.

[72] Rengasamy K.R., Amoo S.O., Aremu A.O., Stirk W.A., Gruz J., Šubrtová M., Doležal K., Van Staden J. (2015). Phenolic profiles, antioxidant capacity, and acetylcholinesterase inhibitory activity of eight south african seaweeds. J. Appl. Phycol..

[73] Lever J., Brkljaca R., Kraft G., Urban S. (2020). Natural products of marine macroalgae from south eastern australia, with emphasis on the port phillip bay and heads regions of victoria. Mar. Drugs..

[74] Saunders G.W.M., Tanya E. (2013). Refinements for the amplification and sequencing of red algal DNA barcode and redtol phylogenetic markers: A summary of current primers, profiles and strategies. Algae.

[75] Heffernan N., Smyth T.J., Soler-Villa A., Fitzgerald R.J., Brunton N.P. (2015). Phenolic content and antioxidant activity of fractions obtained from selected irish macroalgae species (laminaria digitata, fucus serratus, gracilaria gracilis and codium fragile). J. Appl. Phycol..

[76] Leyton A., Pezoa-Conte R., Barriga A., Buschmann A.H., Maki-Arvela P., Mikkola J.P., Lienqueo M.E. (2016). Identification and efficient extraction method of phlorotannins from the brown seaweed macrocystis pyrifera using an orthogonal experimental design. Algal Res..

[77] Feng Y., Dunshea F.R., Suleria H.A.R. (2020). Lc-esi-qtof/ms characterization of bioactive compounds from black spices and their potential antioxidant activities. J. Food Sci. Technol..

[78] Rebaya A., Belghith S.I., Baghdikian B., Leddet V.M., Mabrouki F., Olivier E., Cherif J., Ayadi M.T. (2014). Total phenolic, total flavonoid, tannin content, and antioxidant capacity of halimium halimifolium (cistaceae). J. Appl. Pharm. Sci..

[79] Matanjun P., Mohamed S., Mustapha N.M., Muhammad K., Ming C.H. (2008). Antioxidant activities and phenolics content of eight species of seaweeds from north borneo. J. Appl. Phycol..

