# Supplementary material for: LC-ESI-QTOF-MS/MS Characterization of Seaweed Phenolics and Their Antioxidant Potential
Source: Mar Drugs. 2020 Jun 24;18(6):331. doi: 10.3390/md18060331 (PMC7344666; doi:10.3390/md18060331)
Supplement: Supplementary file 1 [file marinedrugs-18-00331-s001.pdf]

marine drugs

Supplemental material

LC-ESI-QTOF-MS/MS Characterization of seaweed phenolics and their antioxidant potential

Biming Zhong[1], Nicholas A. Robinson[2,3], Robyn D. Warner[1], Colin J. Barrow[4], Frank R. Dunshea[1] and Hafiz A.R. Suleria[1,4*]

1 School of Agriculture and Food, Faculty of Veterinary and Agricultural Sciences, The University of Melbourne, Parkville, VIC 3010, Australia; bimingz@student.unimelb.edu.au (B.Z.); fdunshea@unimelb.edu.au (F.R.D.); robyn.warner@unimelb.edu.au (R.D.W.); hafiz.suleria@unimelb.edu.au (H.A.R.S)
2 Sustainable Aquaculture Laboratory - Temperate and Tropical (SALTT), School of BioSciences, The University of Melbourne, Parkville, VIC 3010, Australia; nicholas.robinson@nofima.no
3 Norwegian Institute of Food, Fisheries and Aquaculture Research (Nofima), NO-1431 Ås, Norway.
4 Centre for Chemistry and Biotechnology, School of Life and Environmental Sciences, Deakin University, Waurn Ponds, VIC 3217, Australia; colin.barrow@deakin.edu.au
* Correspondence: hafiz.suleria@unimelb.edu.au; Tel.: +61 470 439 670

Received: date; Accepted: date; Published: date

Abstract: Seaweed is an important food widely consumed in Asian countries. Seaweed has a diverse array of bioactive compounds, including dietary fiber, carbohydrate, protein, fatty acid, minerals and polyphenols, which contribute to the health benefits and commercial value of seaweed. Nevertheless, detailed information on polyphenol content in seaweeds is still limited. Therefore, the present work aimed to investigate the phenolic compounds present in eight seaweeds [Chlorophyta (green), *Ulva* sp., *Caulerpa* sp. and *Codium* sp.; Rhodophyta (red), *Dasya* sp., *Grateloupia* sp. and *Centroceras* sp.; Ochrophyta (brown), *Ecklonia* sp., *Sargassum* sp.], using liquid chromatography electrospray ionization quadrupole time-of-flight mass spectrometry (LC-ESI-QTOF-MS/MS). The total phenolic content (TPC), total flavonoid content (TFC) and total tannin content (TTC) were determined. The antioxidant potential of seaweed was assessed using a 2,2-diphenyl-1-picrylhydrazyl (DPPH) free radical scavenging assay, a 2,2′-azino-bis-3-ethylbenzothiazoline-6-sulfonic acid (ABTS) free radical scavenging assay and a ferric reducing antioxidant power (FRAP) assay. Brown seaweed species showed the highest total polyphenol content, which correlated with the highest antioxidant potential. The LC-ESI-QTOF-MS/MS tentatively identified a total of 54 phenolic compounds present in the eight seaweeds. Using high-performance liquid chromatography-photodiode array (HPLC-PDA) quantification, the most abundant phenolic compound was *p*-hydroxybenzoic acid, present in *Ulva* sp. at 846.083 ± 0.02 μg/g fresh weight. The results obtained indicate the importance of seaweed as a promising source of polyphenols with antioxidant properties, consistent with the health potential of seaweed in food, pharmaceutical and nutraceutical applications.

Keywords: Seaweeds; polyphenols; antioxidant potential; LC-ESI-QTOF-MS/MS; HPLC-PDA

Table S1: Characterization of phenolic compounds in seaweeds by using LC-ESI-QTOF-MS/MS

No.	Proposed compounds	Molecular Formula	RT (min)	Ionization (ESI^+/ESI^-)	Molecular Weight	Theoretical (*m/z*)	Observed (*m/z*)	Mass Error (ppm)	MS/MS Product ions	Seaweeds
**Phenolic acid**										
**Hydroxybenzoic acids**										
1	Vanillic acid 4-sulfate	$C_8H_8O_7S$	9.112	$[M-H]^-$	247.9991	246.9918	246.9925	2.83	217, 203, 167	**Sargassum* sp., *Centroceras* sp., *Ulva* sp.
2	Gallic acid	$C_7H_6O_5$	9.885	**$[M-H]^-$	170.0215	169.0142	169.0138	-2.37	125	*Centroceras* sp.
3	4-Hydroxybenzoic acid 4-*O*-glucoside	$C_{13}H_{16}O_8$	11.515	$[M-H]^-$	300.0845	299.0772	299.0778	2.01	255, 137	*Sargassum* sp.
4	Protocatechuic acid 4-*O*-glucoside	$C_{13}H_{16}O_9$	13.546	**$[M-H]^-$	316.0794	315.0721	315.0719	-0.63	153	**Centroceras* sp., *Grateloupia* sp.
5	*p*-Hydroxybenzoic acid	$C_7H_6O_3$	27.516	$[M-H]^-$	138.0317	137.0244	137.0240	-2.91	93	**Caulerpa* sp., *Ulva* sp., *Centroceras* sp.
6	Ellagic acid glucoside	$C_{20}H_{16}O_{13}$	38.451	$[M-H]^-$	464.0591	463.0518	463.0518	0.01	301	*Ecklonia* sp.
**Hydroxycinnamic acids**										
7	3-Sinapoylquinic acid	$C_{18}H_{22}O_{10}$	7.005	**$[M-H]^-$	398.1213	397.1140	397.1144	1.01	223, 179	**Centroceras* sp., *Ecklonia* sp.
8	Cinnamoyl glucose	$C_{15}H_{18}O_7$	8.861	**$[M-H]^-$	310.1053	309.098	309.0992	3.88	147, 131, 103	**Codium* sp., *Ulva* sp.
9	Caffeoyl glucose	$C_{15}H_{18}O_9$	10.983	**$[M-H]^-$	342.0951	341.0878	341.0882	1.17	179, 161	**Ecklonia* sp., *Centroceras* sp.
10	Caffeic acid 3-*O*-glucuronide	$C_{15}H_{16}O_{10}$	14.259	**$[M-H]^-$	356.0743	355.0670	355.0671	0.28	179	*Caulerpa* sp.
11	Chlorogenic acid	$C_{16}H_{18}O_9$	15.004	**$[M-H]^-$	354.0951	353.0878	353.0862	-4.53	253, 190, 144	**Centroceras* sp., *Caulerpa* sp.
12	Caffeic acid	$C_9H_8O_4$	18.274	$[M-H]^-$	180.0423	179.0350	179.0350	0.01	151, 143, 133	*Caulerpa* sp.
13	Caffeic acid 4-sulfate	$C_9H_8O_7S$	18.291	$[M-H]^-$	259.9991	258.9918	258.9929	4.25	215, 179, 135	*Caulerpa* sp.
14	Caffeoyl tartaric acid	$C_{13}H_{12}O_9$	24.061	**$[M-H]^-$	312.0481	311.0408	311.0403	-1.61	161	**Grateloupia* sp., *Centroceras* sp.
15	Isoferulic acid 3-sulfate	$C_{10}H_{10}O_7S$	24.520	**$[M-H]^-$	274.0147	273.0074	273.0086	4.4	193, 149	*Caulerpa* sp.
16	Sinapic acid	$C_{11}H_{12}O_5$	25.852	**$[M-H]^-$	224.0685	223.0612	223.0621	4.03	205, 179, 163	**Ulva* sp., *Caulerpa* sp., *Grateloupia* sp.
17	Ferulic acid	$C_{10}H_{10}O_4$	32.604	$[M-H]^-$	194.0579	193.0506	193.0513	3.63	178, 149, 134	*Caulerpa* sp.
18	Coumaric acid	$C_9H_8O_3$	33.797	**$[M-H]^-$	164.0473	163.0400	163.0406	3.68	119	**Ulva* sp., *Ecklonia* sp.
19	Sinapine	$C_{16}H_{24}NO_5$	88.066	$[M+H]^+$	310.1652	310.1654	310.1646	-2.58	251, 207, 175	*Codium* sp.
**Hydroxyphenylpentanoic acids**										
20	5-(3',5'-dihydroxyphenyl)-γ-valerolactone 3-*O*-glucuronide	$C_{17}H_{20}O_{10}$	14.855	**$[M-H]^-$	384.1056	383.0983	383.1001	4.70	221, 206, 191	**Ecklonia* sp., *Codium* sp.
21	5-(3',4'-dihydroxyphenyl)-valeric acid	$C_{11}H_{14}O_4$	51.563	**$[M-H]^-$	210.0892	209.0819	209.0821	0.96	165, 150	*Caulerpa* sp.

**Hydroxyphenylacetic acids**										
22	2-Hydroxy-2-phenylacetic acid	$C_8H_8O_3$	6.18	**$[M+H]^+$	152.0473	153.0546	153.055	2.61	125	**Centroceras* sp., *Caulerpa* sp., *Sargassum* sp.
**Flavonoids**										
**Anthocyanins**										
23	Delphinidin 3-*O*-sambubioside	$C_{26}H_{29}O_{16}$	9.327	$[M+H]^+$	597.1464	597.1456	597.1473	2.85	303, 257, 229	*Grateloupia* sp.
24	Isopeonidin 3-*O*-arabinoside	$C_{21}H_{21}O_{10}$	41.658	$[M+H]^+$	433.1134	433.1135	433.1136	0.23	271, 253, 243	*Centroceras* sp.
25	Malvidin 3-*O*-glucoside	$C_{23}H_{25}O_{12}$	54.152	$[M+H]^+$	493.1343	493.1346	493.1343	-0.61	331	*Centroceras* sp.
**Flavanols**										
26	Gallocatechin	$C_{15}H_{14}O_7$	7.604	**$[M-H]^-$	306.0740	305.0667	305.0668	0.33	261, 219	**Caulerpa* sp., *Ulva* sp., *Dasya* sp., *Ecklonia* sp., *Sargassum* sp.
27	3'-*O*-Methylcatechin	$C_{16}H_{16}O_6$	17.857	**$[M-H]^-$	304.0947	303.0874	303.0886	3.96	271, 163	*Grateloupia* sp.
28	Catechin (isomer)	$C_{15}H_{14}O_6$	45.118	$[M-H]^-$	290.0790	289.0717	289.0731	4.84	245, 205, 179	*Caulerpa* sp.
**Flavonols**										
29	Quercetin 3-*O*-(6”-malonyl-glucoside)	$C_{24}H_{22}O_{15}$	9.902	$[M-H]^-$	550.0959	549.0886	549.0887	0.18	463, 301, 161	*Centroceras sp., Caulerpa sp.
30	5,3',4'-Trihydroxy-3-methoxy-6:7-methylenedioxyflavone 4'-*O*-glucuronide	$C_{23}H_{20}O_{14}$	33.878	$[M-H]^-$	520.0853	519.0780	519.0779	-0.19	343	*Ecklonia* sp.
31	3,7-Dimethylquercetin	$C_{17}H_{14}O_7$	80.642	$[M-H]^-$	330.0740	329.0667	329.0674	2.13	314, 299, 271	*Centroceras* sp.
**Flavones**										
32	Rhoifolin	$C_{27}H_{30}O_{14}$	44.036	**$[M-H]^-$	578.1636	577.1563	577.1588	4.33	413, 269	*Centroceras* sp.
**Isoflavonoids**										
33	Sativanone	$C_{17}H_{16}O_5$	4.240	$[M-H]^-$	300.0998	299.0925	299.0918	-2.34	284, 269, 225	*Ecklonia* sp.
34	Glycitein 7-*O*-glucuronide	$C_{22}H_{20}O_{11}$	4.454	**$[M-H]^-$	460.1006	459.0933	459.0923	-2.18	283, 268, 117	*Centroceras* sp.
35	3',4',5,7-Tetrahydroxyisoflavanone	$C_{15}H_{12}O_6$	4.640	**$[M-H]^-$	288.0634	287.0561	287.0556	-1.74	269, 259	**Caulerpa* sp., *Grateloupia* sp., *Centroceras* sp.
36	3'-*O*-Methylequol	$C_{16}H_{16}O_4$	4.803	**$[M-H]^-$	272.1049	271.0976	271.0972	-1.48	147, 123, 121	**Ecklonia* sp., *Grateloupia* sp.
37	Dalbergin	$C_{16}H_{12}O_4$	9.344	**$[M-H]^-$	268.0736	267.0663	267.0666	1.12	252, 224, 180	**Grateloupia* sp., *Centroceras* sp.
38	Dihydrobiochanin A	$C_{16}H_{14}O_5$	80.715	**$[M-H]^-$	286.0841	285.0768	285.0771	1.05	270	**Codium* sp., *Centroceras* sp.
39	3'-Hydroxydaidzein	$C_{15}H_{10}O_5$	86.956	$[M-H]^-$	270.0528	269.0455	269.0457	0.74	151, 117, 107	**Grateloupia* sp., *Centroceras* sp., *Caulerpa* sp., *Ecklonia* sp.
**Other polyphenols**										
**Hydroxybenzaldehydes**										

40	*p*-Hydroxybenzaldehyde	$C_7H_6O_2$	15.921	$[M-H]^-$	122.0368	121.0295	121.0295	0.01	92, 77	**Dasya* sp., *Ecklonia* sp., *Codium* sp.
**Hydroxycoumarins**										
41	Urolithin A	$C_{13}H_8O_4$	4.64	$[M-H]^-$	228.0423	227.0350	227.0341	-3.96	198, 182	*Grateloupia* sp.
42	Scopoletin	$C_{10}H_8O_4$	84.705	**$[M-H]^-$	192.0423	191.0350	191.0352	1.05	176, 147	**Codium* sp., *Grateloupia* sp., *Sargassum* sp.
**Phenolic terpenes**										
43	Rosmanol	$C_{20}H_{26}O_5$	24.965	$[M+H]^+$	346.1780	347.1853	347.1843	-2.88	301, 231	**Dasya* sp., *Ulva* sp., *Grateloupia* sp., *Ecklonia* sp., *Codium* sp.
44	Carnosol	$C_{20}H_{26}O_4$	85.931	**$[M-H]^-$	330.1831	329.1758	329.1747	-3.34	287, 286, 285	**Codium* sp., *Caulerpa* sp.
45	Carnosic acid	$C_{20}H_{28}O_4$	86.958	**$[M-H]^-$	332.1988	331.1915	331.1912	-0.91	287, 269	**Ecklonia* sp., *Dasya* sp., *Codium* sp., *Sargassum* sp.
**Tyrosols**										
46	Hydroxytyrosol 4-*O*-glucoside	$C_{14}H_{20}O_8$	36.653	**$[M-H]^-$	316.1158	315.1085	315.1091	1.90	153, 123	**Centroceras* sp., *Dasya* sp., *Grateloupia* sp., *Sargassum* sp.
47	3,4-DHPEA-EDA	$C_{17}H_{20}O_6$	87.423	$[M-H]^-$	320.1260	319.1187	319.1200	4.07	301, 275, 195	*Caulerpa* sp.
**Other polyphenols**										
48	3,4-Dihydroxyphenylglycol	$C_8H_{10}O_4$	7.005	$[M-H]^-$	170.0579	169.0506	169.0503	-1.77	141, 139, 123	*Centroceras* sp.
49	Phloroglucinol	$C_6H_6O_3$	14.793	$[M-H]^-$	126.0317	125.0244	125.0242	-1.59	97	**Ecklonia* sp., *Sargassum* sp.
50	Isopropyl 3-(3,4-dihydroxyphenyl)-2-hydroxypropanoate	$C_{12}H_{16}O_5$	24.882	**$[M-H]^-$	240.0998	239.0925	239.0919	-2.51	195, 155, 99	*Dasya* sp.
**Lignans**										
**Lignans**										
51	2'-Hydroxyenterolactone	$C_{18}H_{18}O_5$	7.781	$[M-H]^-$	314.1154	313.1081	313.1082	0.32	295, 283	*Grateloupia* sp.
52	Arctigenin	$C_{21}H_{24}O_6$	8.131	**$[M-H]^-$	372.1573	371.1500	371.1509	2.42	356, 312, 295	**Centroceras* sp., *Sargassum* sp.
53	Dimethylmatairesinol	$C_{22}H_{26}O_6$	83.663	$[M+H]^+$	386.1729	387.1802	387.1805	0.77	372, 369, 357, 329	**Caulerpa* sp., *Dasya* sp.
54	Deoxyschisandrin	$C_{24}H_{32}O_6$	85.152	**$[M+H]^+$	416.2199	417.2272	417.2286	3.36	402, 347, 316, 301	**Ecklonia* sp., *Codium* sp., *Sargassum* sp.

* Compound was detected in more than one seaweed samples, data presented in this table are from single asterisk sample. ** Compounds were detected in both negative $[M-H]^-$ and positive $[M+H]^+$ mode of ionization while single mode data was presented. RT stands for retention time.

(1a)

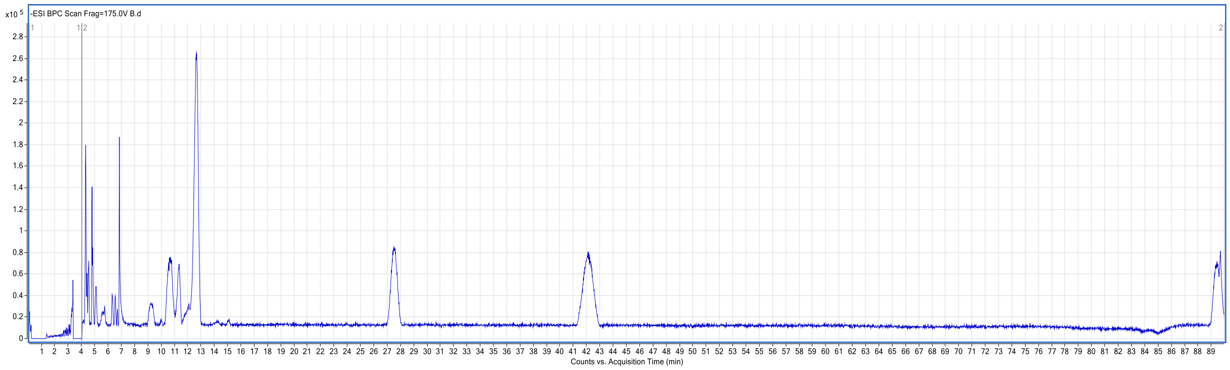
-ESI BPC Scan Frag=175.0V B.d
Counts vs. Acquisition Time (min)

(1b)

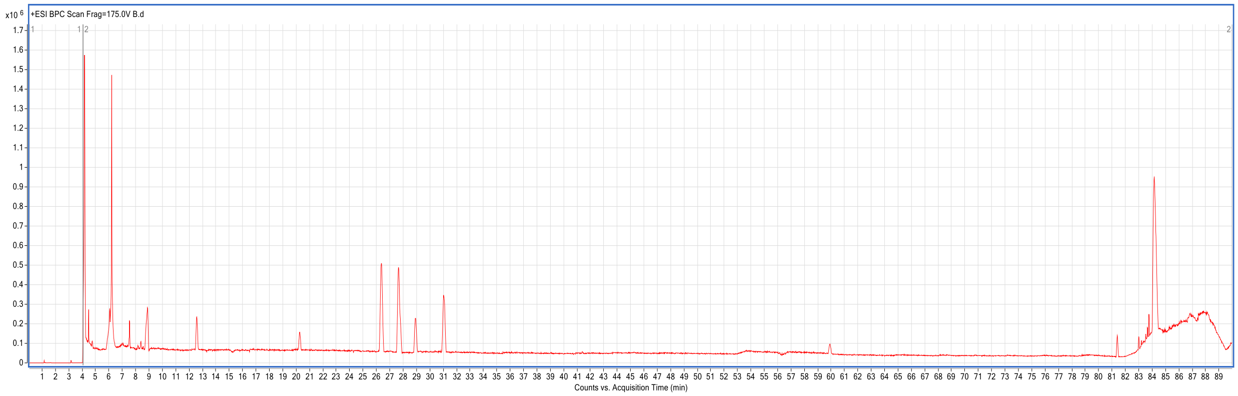
+ESI BPC Scan Frag=175.0V B.d
Counts vs. Acquisition Time (min)

(2a)

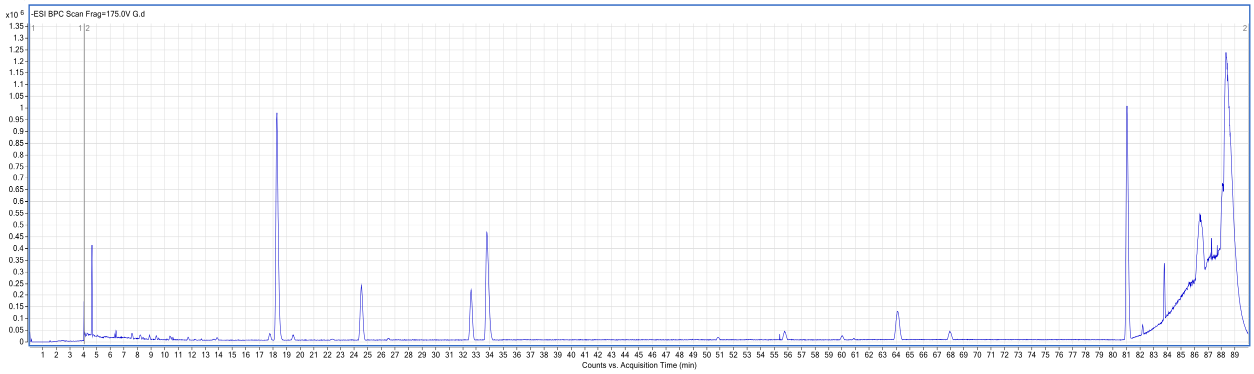
-ESI BPC Scan Frag=175.0V G.d
Counts vs. Acquisition Time (min)

(2b)

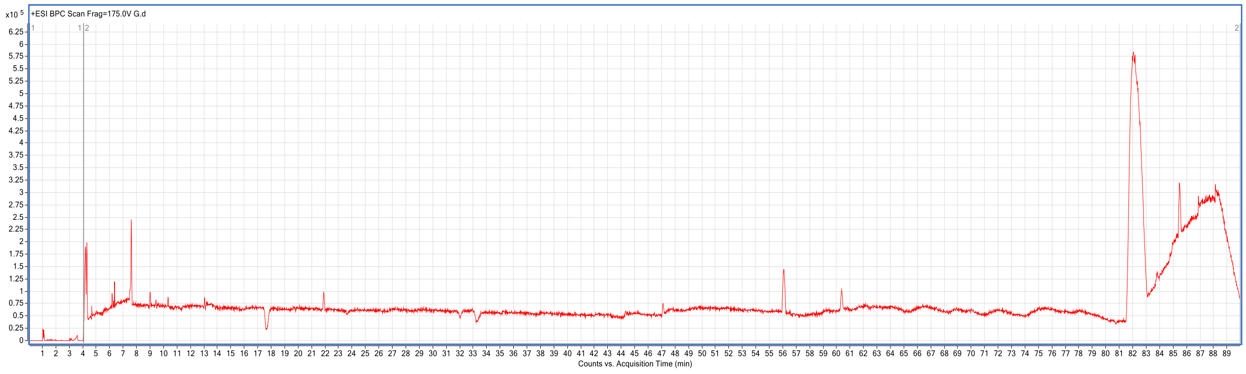
+ESI BPC Scan Frag=175.0V G.d
Counts vs. Acquisition Time (min)

(3a)

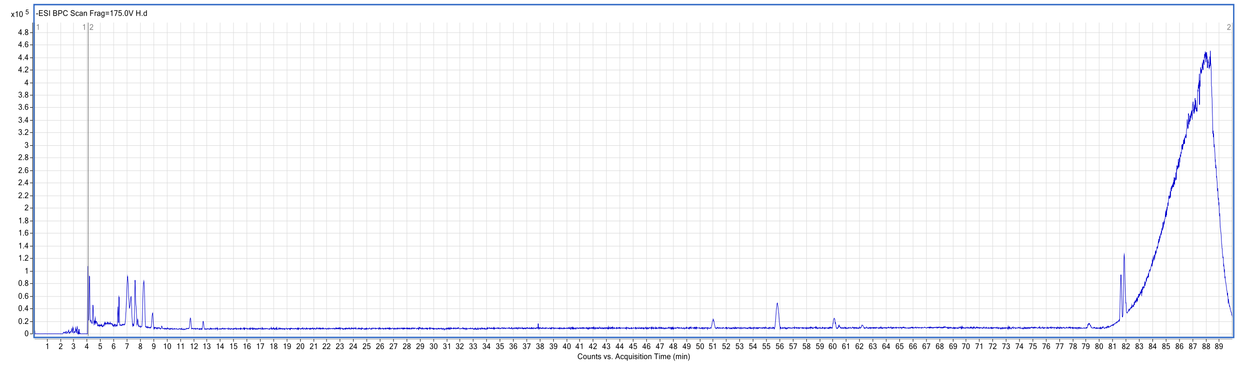
-ESI BPC Scan Frag=175.0V H.d
Counts vs. Acquisition Time (min)

(3b)

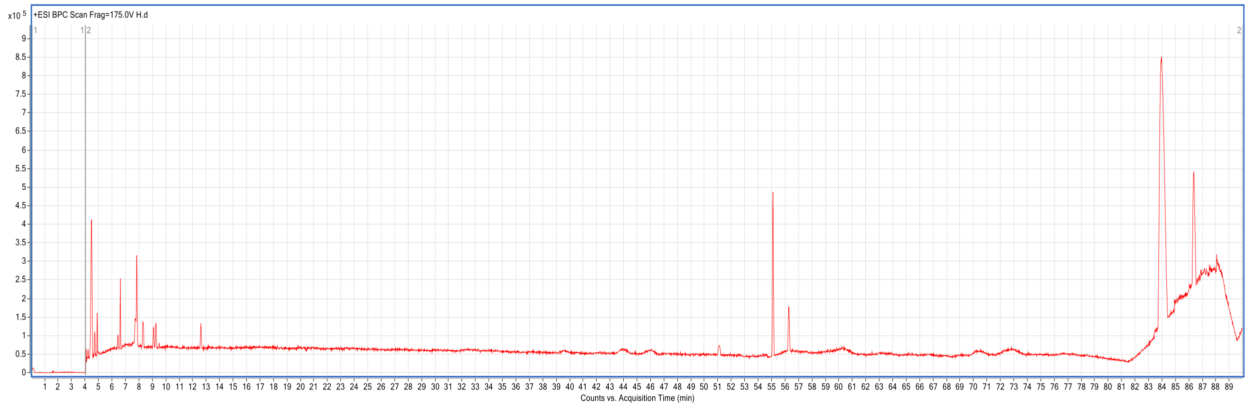
+ESI BPC Scan Frag=175.0V H.d
Counts vs. Acquisition Time (min)

(4a)

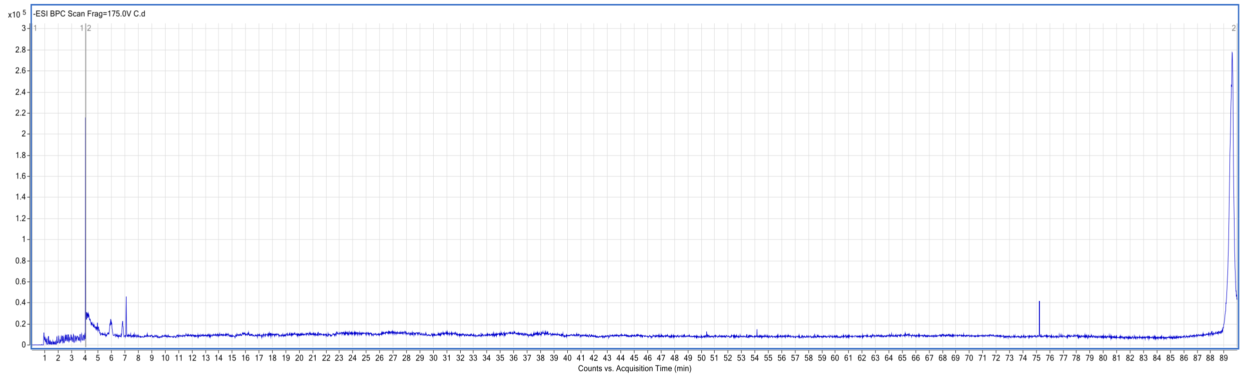
-ESI BPC Scan Frag=175.0V C.d
Counts vs. Acquisition Time (min)

(4b)

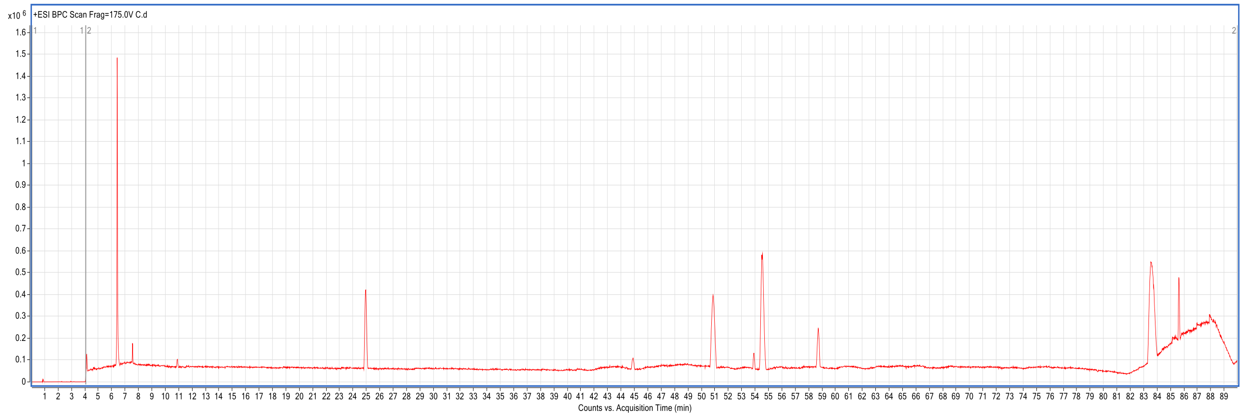
+ESI BPC Scan Frag=175.0V C.d
Counts vs. Acquisition Time (min)

(5a)

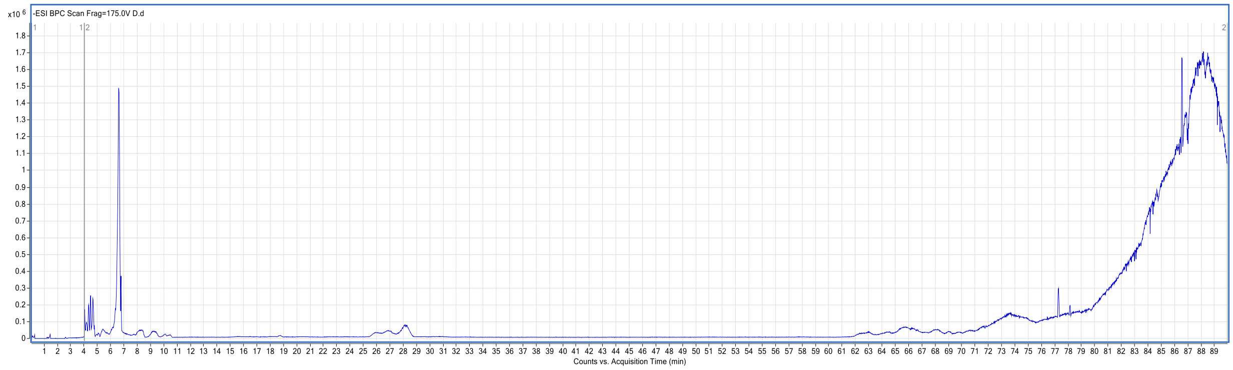
x10 6
-ESI BPC Scan Frag=175.0V D.d
Counts vs. Acquisition Time (min)

(5b)

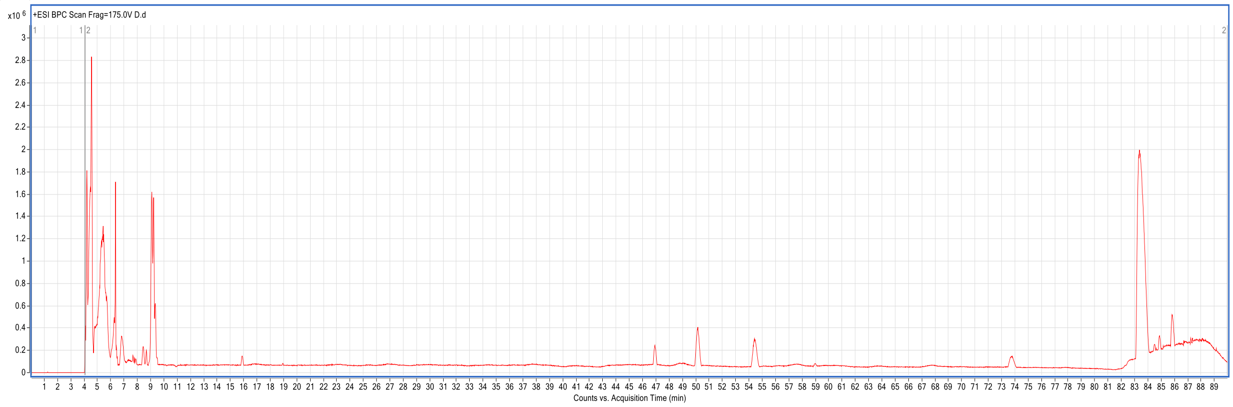
x10 6
+ESI BPC Scan Frag=175.0V D.d
Counts vs. Acquisition Time (min)

(6a)

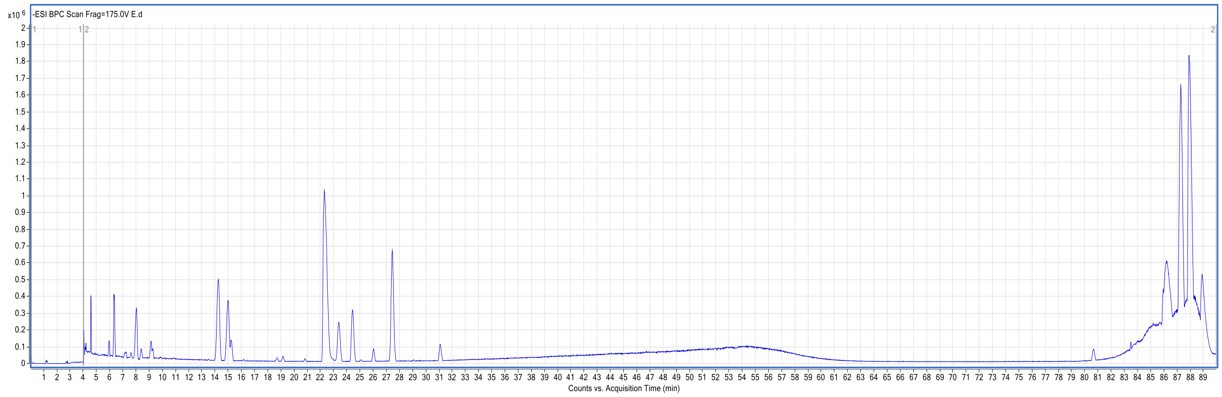
x10 6
-ESI BPC Scan Frag=175.0V E.d
Counts vs. Acquisition Time (min)

(6b)

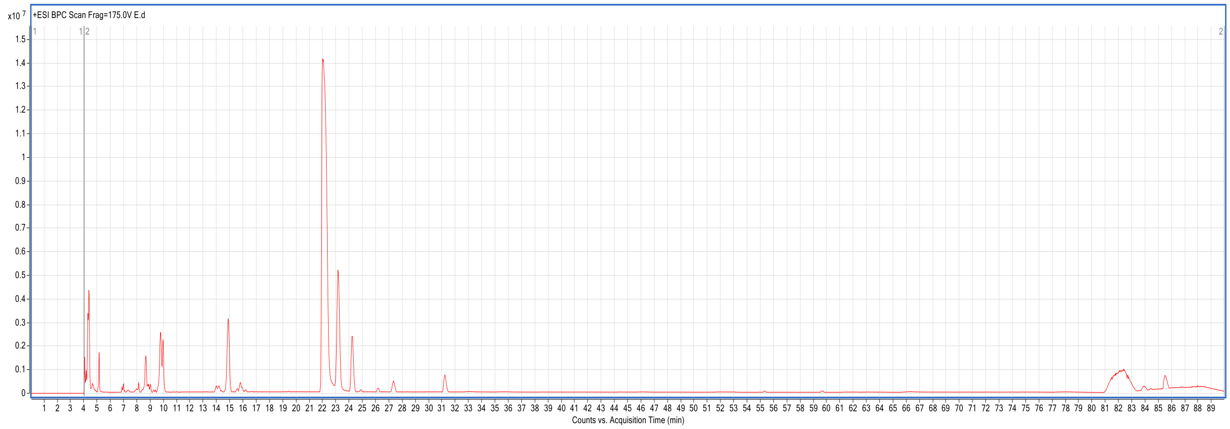
x10 7
+ESI BPC Scan Frag=175.0V E.d
Counts vs. Acquisition Time (min)

(7a)

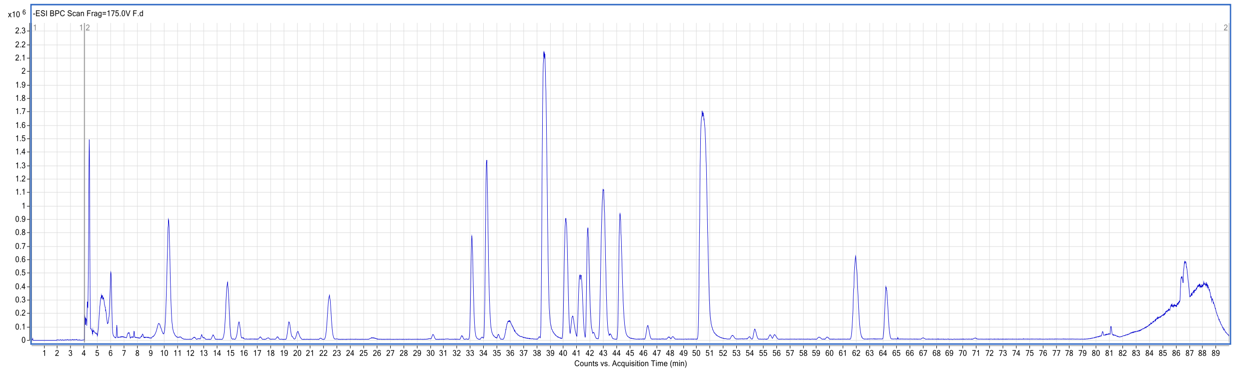
x10 6
-ESI BPC Scan Frag=175.0V F.d
Counts vs. Acquisition Time (min)

(7b)

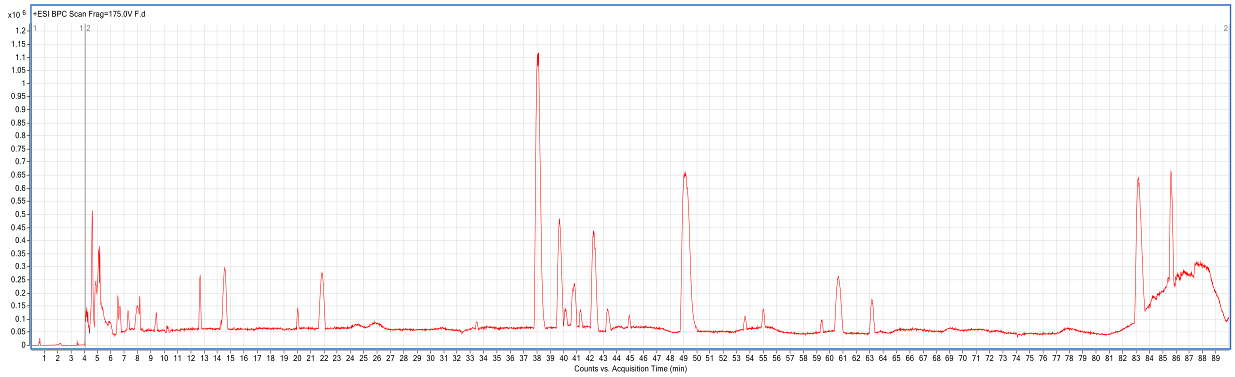
x10 6
+ESI BPC Scan Frag=175.0V F.d
Counts vs. Acquisition Time (min)

(8a)

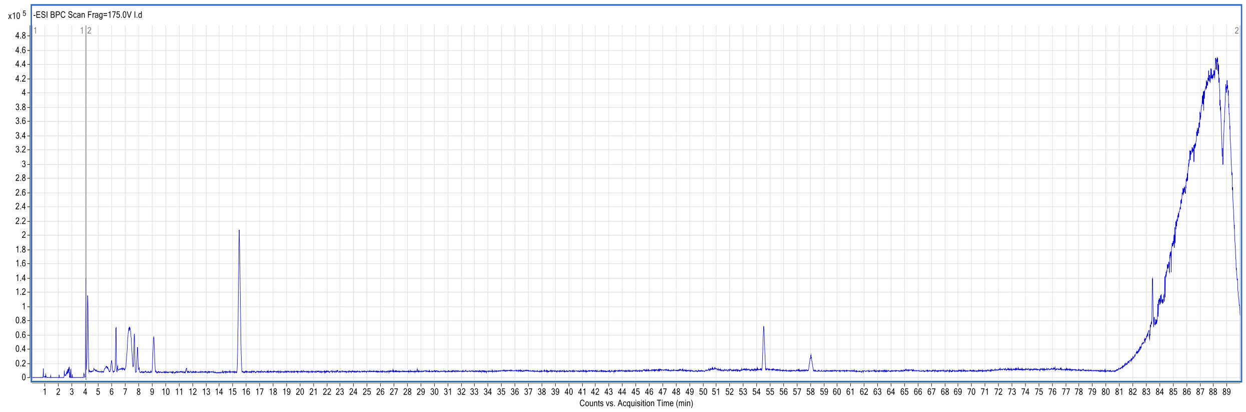
x10 5
-ESI BPC Scan Frag=175.0V I.d
Counts vs. Acquisition Time (min)

(8b)

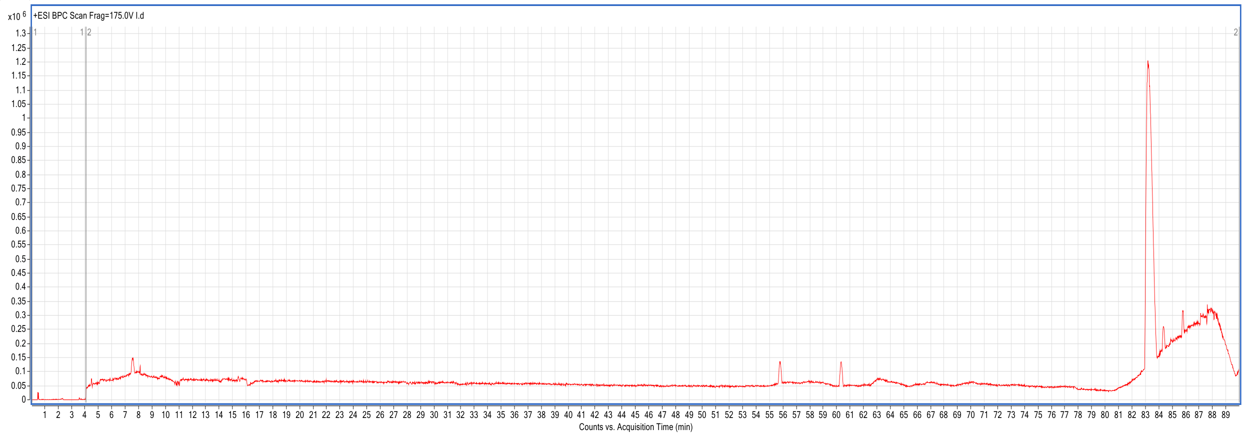
x10 6
+ESI BPC Scan Frag=175.0V I.d
Counts vs. Acquisition Time (min)

Figure (S1): Base peak chromatogram (BPC) for characterization of phenolic compounds of seaweeds. (1a) *Ulva* sp. BPC in negative ionization mode; **(1b)** *Ulva* sp. BPC in positive ionization mode; **(2a)** *Caulerpa* sp. BPC in negative ionization mode; **(2b)** *Caulerpa* sp. BPC in positive ionization mode; **(3a)** *Codium* sp. BPC in negative ionization mode; **(3b)** *Codium* sp. BPC in positive ionization mode; **(4a)** *Dasya* sp. BPC in negative ionization mode; **(4b)** *Dasya* sp. BPC in positive ionization mode; **(5a)** *Grateloupia* sp. BPC in negative ionization mode; **(5b)** *Grateloupia* sp. BPC in positive ionization mode; **(6a)** *Centroceras* sp. BPC in negative ionization mode; **(6b)** *Centroceras* sp. BPC in positive ionization mode; **(7a)** *Ecklonia* sp. BPC in negative ionization mode; **(7b)** *Ecklonia* sp. BPC in positive ionization mode; **(8a)** *Sargassum* sp. BPC in negative ionization mode; **(8b)** *Sargassum* sp. BPC in positive ionization mode.

(a)

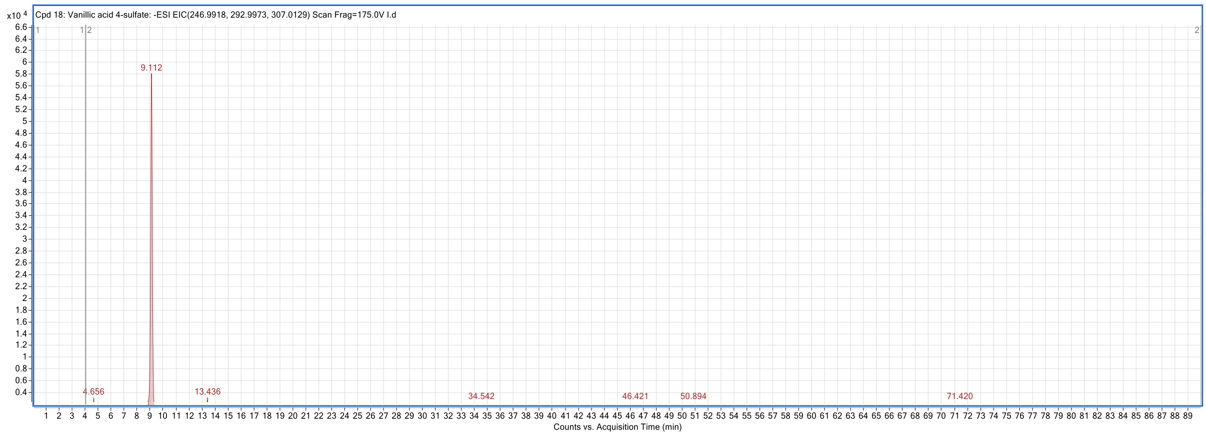
Cpd 18: Vanillic acid 4-sulfate: -ESI EIC(246.9918, 292.9973, 307.0129) Scan Frag=175.0V I.d
x10 4
9.112
4.656
13.436
34.542
46.421
50.894
71.420
Counts vs. Acquisition Time (min)

(b)

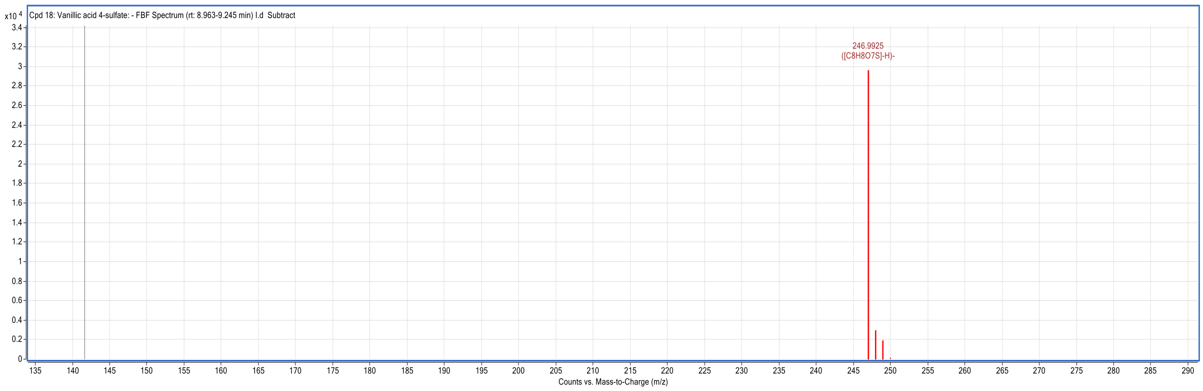
Cpd 18: Vanillic acid 4-sulfate: - FBF Spectrum (rt: 8.963-9.245 min) I.d Subtract
x10 4
246.9925
([C8H8O7S]-H)-
Counts vs. Mass-to-Charge (m/z)

(c)

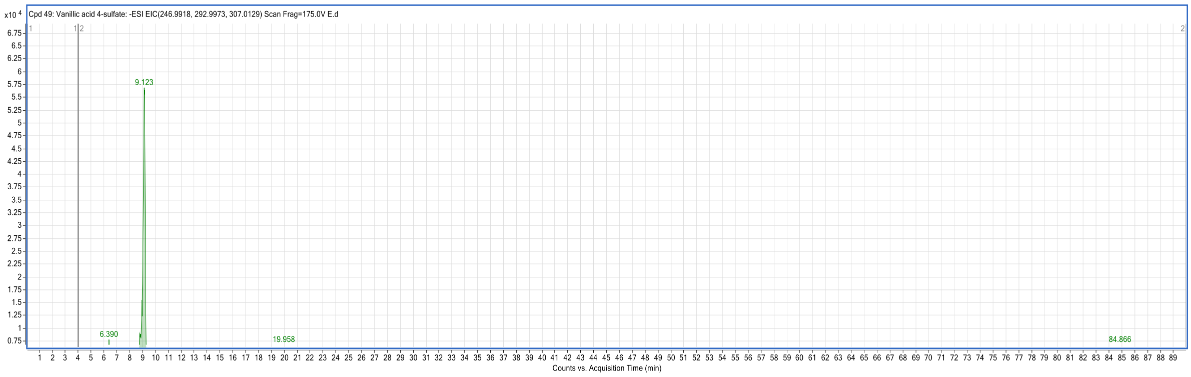
Cpd 49: Vanillic acid 4-sulfate: -ESI EIC(246.9918, 292.9973, 307.0129) Scan Frag=175.0V E.d
x10 4
9.123
6.390
19.958
84.866
Counts vs. Acquisition Time (min)

(d)

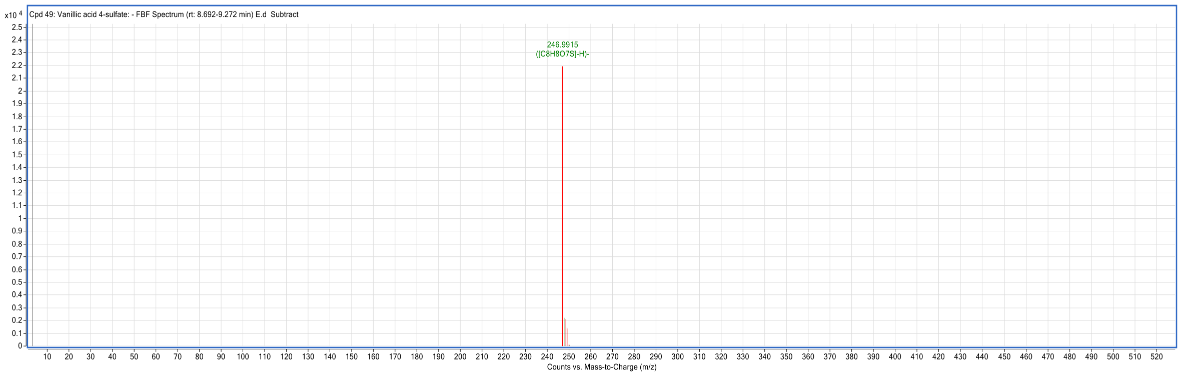
Cpd 49: Vanillic acid 4-sulfate: - FBF Spectrum (rt: 8.692-9.272 min) E.d Subtract
x10 4
246.9915
([C8H8O7S]-H)-
Counts vs. Mass-to-Charge (m/z)

(e)

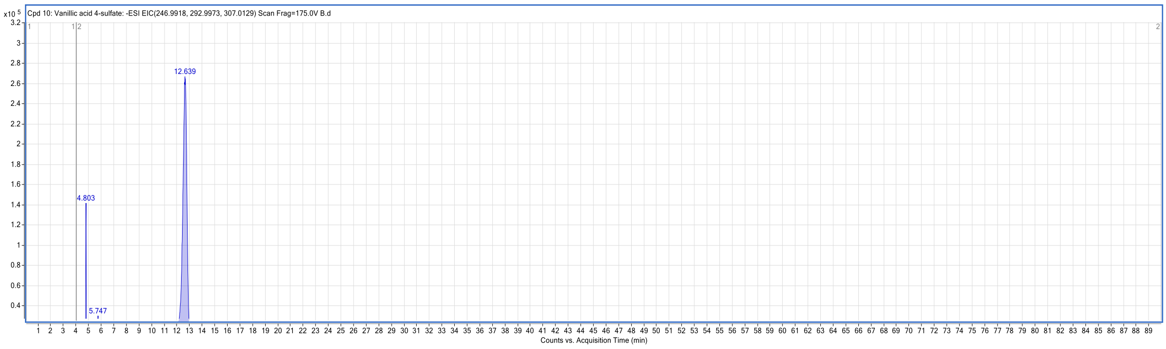

(f)

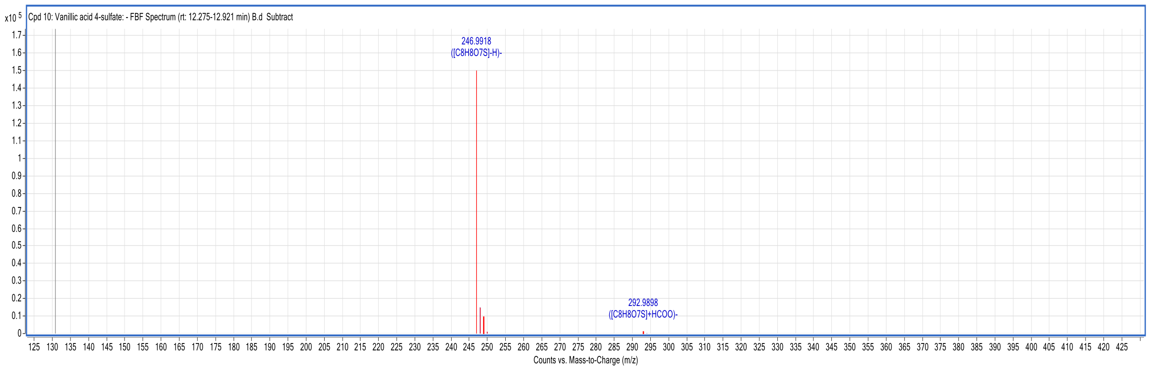

Figure (S2). Extracted ion chromatogram and mass spectrum of "Vanillic acid 4-sulfate" detected in three different seaweeds. (a) A chromatograph of vanillic acid 4-sulfate (Compound **1**, Table 1S) identified in *Sargassum* sp. in negative mode of ionization (ESI$^-$/[M-H]$^-$); **(b)** Mass spectra of vanillic acid 4-sulfate showing an observed *m/z* 246.9925 in *Sargassum* sp. **(c)** A chromatograph of vanillic acid 4-sulfate identified in *Centroceras* sp. in negative mode; **(d)** Mass spectra of vanillic acid 4-sulfate showing an observed *m/z* 246.9915 in *Centroceras* sp.; **(e)** A chromatograph of vanillic acid 4-sulfate identified in *Ulva* sp. in negative mode; **(f)** Mass spectra of vanillic acid 4-sulfate showing an observed *m/z* 246.9918 in *Ulva* sp.
